# Theta Phase Entrainment of Single-Cell Spiking in Rat Somatosensory Barrel Cortex and Secondary Visual Cortex Is Enhanced during Multisensory Discrimination Behavior

**DOI:** 10.1523/ENEURO.0180-23.2024

**Published:** 2024-04-24

**Authors:** Thijs R. Ruikes, Julien Fiorilli, Judith Lim, Gerjan Huis in ‘t Veld, Conrado Bosman, Cyriel M. A. Pennartz

**Affiliations:** Center for Neuroscience, Faculty of Science, Swammerdam Institute for Life Sciences, University of Amsterdam, Amsterdam 1098 XH, The Netherlands

**Keywords:** barrel cortex, hippocampus, multisensory discrimination, rat behavior, theta phase entrainment, visual cortex

## Abstract

Phase entrainment of cells by theta oscillations is thought to globally coordinate the activity of cell assemblies across different structures, such as the hippocampus and neocortex. This coordination is likely required for optimal processing of sensory input during recognition and decision-making processes. In quadruple-area ensemble recordings from male rats engaged in a multisensory discrimination task, we investigated phase entrainment of cells by theta oscillations in areas along the corticohippocampal hierarchy: somatosensory barrel cortex (S1BF), secondary visual cortex (V2L), perirhinal cortex (PER), and dorsal hippocampus (dHC). Rats discriminated between two 3D objects presented in tactile-only, visual-only, or both tactile and visual modalities. During task engagement, S1BF, V2L, PER, and dHC LFP signals showed coherent theta-band activity. We found phase entrainment of single-cell spiking activity to locally recorded as well as hippocampal theta activity in S1BF, V2L, PER, and dHC. While phase entrainment of hippocampal spikes to local theta oscillations occurred during sustained epochs of task trials and was nonselective for behavior and modality, somatosensory and visual cortical cells were only phase entrained during stimulus presentation, mainly in their preferred modality (S1BF, tactile; V2L, visual), with subsets of cells selectively phase-entrained during cross-modal stimulus presentation (S1BF: visual; V2L: tactile). This effect could not be explained by modulations of firing rate or theta amplitude. Thus, hippocampal cells are phase entrained during prolonged epochs, while sensory and perirhinal neurons are selectively entrained during sensory stimulus presentation, providing a brief time window for coordination of activity.

## Significance Statement

Neural activity during theta oscillations (6–12 Hz) has long been considered a mechanism for interareal communication, but its temporal dynamics in relation to sensory and mnemonic processing are still poorly understood. We report how sensory neocortical and hippocampal areas temporally coordinate their activity with local field potential activity in the theta band during a behavioral task involving multisensory object discrimination and recognition. Theta phase entrainment in sensory cortical areas selectively occurred during behavioral task epochs where object information was presented in the preferred stimulus modality of a given area. This entrainment was largely independent of firing rate. These findings support the framework of theta-band synchrony as a mechanism for facilitating corticohippocampal communication during sensory and mnemonic processing.

## Introduction

The rodent hippocampal local field potential (LFP) expresses prominent theta oscillations (6–12 Hz) during awake behaviors such as locomotion (Sinnamon, 2006; Kropff et al., 2021), whisking during object sampling (Grion et al., 2016), orienting behaviors (Whishaw and Vanderwolf, 1973; Buzsáki, 2009), and REM sleep (Buzsáki, 2009; Bandarabadi et al., 2019). Hippocampal theta oscillations are generated through synchronized synaptic input from the medial septal area (Brandon et al., 2014; Quirk et al., 2021) and represent the coordinated activity of neural ensembles (Buzsáki et al., 2012; Einevoll et al., 2013). Such coordination is thought to facilitate memory encoding (read-in) and retrieval (read-out; Battaglia et al., 2011; Colgin, 2013; Lisman et al., 2017; Buzsáki and Tingley, 2018). In addition, phase entrainment of cell firing by theta oscillations may facilitate communication between areas. In contrast to phase coding (e.g., the encoding of an animal's trajectory through a place field by spike timing relative to theta phase; O’Keefe and Recce, 1993; Huxter et al., 2008; Petersen and Buzsáki, 2020; Yiu et al., 2022), phase entrainment does not imply information coding in spike timing but rather suggests a mechanistic function of oscillations in communication and information transmission. The synchronized input of an entraining entity, whether internal (such as the hippocampus) or external (such as sensory input), could bias spike timing in receiver areas (Salinas and Sejnowski, 2000) and thereby increase synchrony within and between areas and improve interareal communication (Bosman et al., 2014; Singer, 2018). To clarify, different definitions of “synchrony” or “synchronization” are used in the literature. Whereas “synchrony” traditionally refers to neural events occurring simultaneously, or without a phase lag in case of periodic signals, we will use the term in the sense of “phase synchronization,” the phenomenon that periodic signals oscillate with a consistent phase angle relative to each other (Vinck et al., 2011).

Evidence for phase-entrainment by theta oscillations has been found in many areas along the corticohippocampal hierarchy, including somatosensory cortex (Grion et al., 2016; Vinck et al., 2016), visual cortex (Vinck et al., 2016), auditory cortex (Parto Dezfouli et al., 2019), and medial temporal lobe areas such as perirhinal (Bos et al., 2017; Ahn et al., 2019), postrhinal (Furtak et al., 2012), and entorhinal cortex (Alonso and García-Austt, 1987), as well as in subcortical regions such as the ventral striatum (Lansink et al., 2016). Phase entrainment of cells throughout the neocortex by theta oscillations might serve as a mechanism to process sensory information (Kleinfeld et al., 2016; Lakatos et al., 2019) and coordinate memory storage and retrieval required for episodic memory between neocortical areas and hippocampus (Csicsvari et al., 1999; Buzsáki, 2009; Battaglia et al., 2011).

Phase entrainment of cortical cells has been mainly studied in the context of spatial processing and only a few studies addressed phase entrainment by theta oscillations during discrete stimulus processing in freely behaving rodents (Grion et al., 2016). As a result, the temporal dynamics of neocortical phase entrainment and its association with particular behaviors elicited by precisely timed sensory stimuli remain largely unclear. Does phase entrainment of cortical cells manifest itself selectively during epochs of heightened sensory processing and object recognition? Furthermore, how does neocortical theta phase entrainment compare with entrainment in the hippocampus along the time course of sensory discrimination trials?

To answer these questions, we performed ensemble recordings in freely behaving rats during a multisensory two-alternative choice task in a T-maze. Our behavioral paradigm consisted of distinct trial epochs: (1) approach to the object sampling port, (2) sensory object discrimination and recognition, (3) approach to a reward site, and (4) trial outcome. During this experiment, we simultaneously recorded neural activity from two sensory neocortical areas (primary somatosensory barrel cortex and secondary visual cortex) and two medial temporal lobe areas (perirhinal cortex and hippocampus).

Our results show phase entrainment of neocortical cells by hippocampal and neocortical theta oscillations during the sensory discrimination and object recognition phase, while these cells were not phase entrained during navigation and consumptive behavior. In perirhinal cortex, phase entrainment was selective for sensory discrimination as well, with some cells selective for the tactile and other cells selective for the visual modality. In contrast, hippocampal ensembles showed theta phase entrainment throughout the behavioral trial, such that different cells tessellated successive task phases. Our findings support a general role for theta oscillations in temporally organizing and synchronizing spiking activity in the hippocampus, whereas the entrainment in the sensory and perirhinal cortices is especially enhanced during the restricted period of sensory object processing.

## Materials and Methods

### Experimental design and statistical analysis

#### Experimental animals

Four male Lister Hooded rats (aged 7–10 months) were housed in pairs during behavioral training. Following implantation, they were individually housed in transparent cages (40 × 40 × 40 cm). All rats were maintained on a reversed 12 h light/dark schedule and tested in the dark phase. During all experiments, rats were food deprived, maintaining body weight of at least 85% of their free-feeding body weight. The experiments were performed in accordance with the National Guidelines on Animal Experiments and were approved by the Animal Experimentation Committee of the University of Amsterdam.

#### Behavioral apparatus

**Figure 1.**
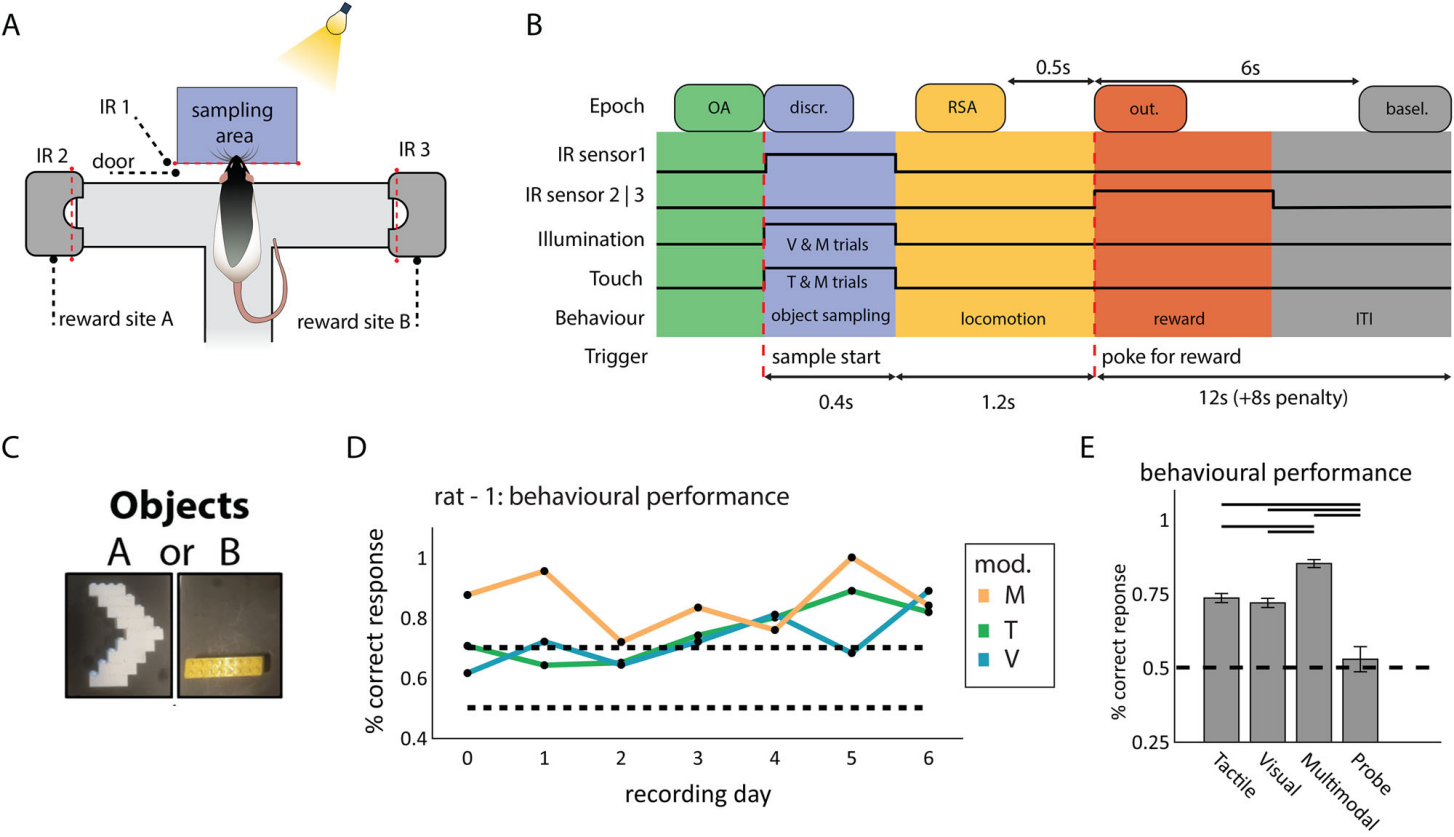
Simultaneous multiarea ensemble recordings during the two-alternative forced choice object discrimination task. ***A***, Overview of the behavioral apparatus. The maze (gray) is situated next to an object sampling area (top, blue) and between two reward sites (left and right arm). Animal movement is tracked using infrared sensors (IR 1, 2, and 3). Tactile stimuli are controlled by moving the object in and out of whisker reach; visual stimuli are controlled by an LED array. ***B***, Temporal layout of the behavioral task. Following the opening of the door in front of the sampling area, the animal could access the presented object. In the sampling area, the animal was presented with one out of two objects, either in the tactile (T, touch only), visual (V, visual only), or multisensory (M, tactile and visual) condition. Following object sampling, the animal navigated either to reward site A or B. Five types of animal behavior are defined: object approach (OA; the animal approaches the sampling area), sensory discrimination of the object (discr.; the animal enters the sampling area and identifies the object), reward site approach (RSA; following exit of the sampling area), outcome (out.; following poke for reward), and ITI (12 s in duration, +8 s if no reward was dispensed) wherefrom the baseline (basel.) data was sampled. Per trial, epochs of 500 ms for these five behavioral epochs were used for analysis. Durations underneath are the average durations of each behavioral epoch across all sessions (the 0.4 s marked under “discr.” pertains to the moment when the animal starts moving away from the object, sampling ends when the animal exits the area, the latter was not tracked). ***C***, Objects to be discriminated during the sampling epoch. ***D***, Animals learned to discriminate the same two objects in different modalities. Example performance across recording days per modality for Rat 1. ***E***, Performance is averaged over the four recorded animals, per modality trial type (error bars denote standard errors of the mean, horizontal bars indicate significant differences between conditions; *p* < 0.01, paired *t* test; dashed horizontal line: chance level).

Behavioral training was performed in a darkened room on a T-shaped, elevated platform [Fig. 1*A*; width 30 (base) × length 60 (arms left to right) × height 60 cm]. A pneumatic door controlled access to the sampling area, the sampling area itself was marked by a gap wherein the rat could only reach with its head, but not any other body parts. The presence of the animal at the sampling area was tracked using an infrared sensor detected by a phototransistor (Fig. 1*A*, IR sensor 1). Similarly, entry of the animal into the reward ports was tracked (Fig. 1*A*, IR sensor 2 and 3). Sucrose solution (15% in water, 60–70 µl) was delivered as reward through fluid wells positioned at the end of the side arms of the maze. Nose pokes by the rat into the well, and licks in the reward well, were tracked using an infrared sensor and phototransistor.

The sampled objects consisted of two duplicated pairs of Lego configurations mounted on a rectangular rotatable platform controlled by a stepper motor (Fig. 1*C*). To control for motor sounds during object rotation, the objects were rotated randomly at the start of the intertrial interval (ITI). Objects mounted on the long side of the platform were within the rat's whisking range and presented during tactile and multisensory trials (∼15–16 cm away from the maze). Objects mounted on the short side of the rectangle were outside the rat's whisking range, behind a 22–23 cm gap, and were presented during visual and probe trials. During visual and multisensory trials, the object was illuminated using white light LEDs.

#### Behavioral paradigm

Each trial of the two-alternative forced choice multisensory discrimination task began with an ITI of 12 s in duration, which was followed by the opening of the door giving access to the sampling area. Rats entered the sampling area, where one out of two objects was presented in the tactile, visual, or multisensory condition. These objects remained the same during the training and experiment period and were familiar to the animal during recording. During the tactile condition the rat could only perceive the object using its whiskers (the experimental setup was in a dark room), during the visual condition the rat could only see, but not touch, the object and during the multisensory condition the rat could sample the object both with its whiskers and by way of visual access. Each of the objects was associated with one response side, that is, the left or right arm of the maze, and if the rat would poke into the fluid well at the correct side, it was rewarded with sucrose solution. Thus, in addition to object discrimination, the task involved object recognition based on which rats had to choose the side arm associated with the object presented in the current trial. For instance, when recognizing object A, the rat had to make a response into the left arm to be rewarded at the left fluid well, whereas the opposite arm had to be chosen after sampling object B. The object-side coupling remained constant across all sessions of a given rat. In case of an incorrect response, the ITI was extended by 8 s. Behavioral training spanned several months and was complete once stable performance of 70% correct for each modality during seven consecutive days was reached.

#### Definition of behavioral epochs

Five types of behavior were defined: baseline behavior, approach, sampling, navigation, and outcome. For each of these epochs, a trigger emitted by the behavioral apparatus was used as reference time point; for analysis we used a 500 ms epoch following and aligned to this trigger to segment the data. The epochs were defined as follows (Fig. 1*B*):

1. Baseline: 6–6.5 s following the nose poke for reward (trigger: IR sensor 2 or IR sensor 3) in the foregoing trial. The animal rests, grooms, or wanders around the platform.
2. Object approach: −0.5 to 0 s before passing IR sensor 1, during which the animal navigates toward the sampling area.
3. Sensory discrimination: 0–0.5 s following the passage of IR sensor 1, which triggered light onset in the sampling area (during visual and multisensory trials) and allowed for tactile palpation with the whiskers of the stimulus (during tactile and multisensory trials). The animal engages in sensory sampling of the object (trials wherein the animal withdrew its head before 200 ms after passing IR sensor 1 were excluded from analysis).
4. Reward site approach: −1 to −0.5 s before the nose poke for reward (trigger: IR sensor 2 or IR sensor 3). The animal chooses a side and navigates to one of the reward locations. One of these wells is associated with the sampled object and is rewarded when chosen.
5. Outcome: 0–0.5 s following the reward poke (trigger: IR sensor 2 or IR sensor 3). The animal pokes into the reward well and receives a reward in case of a correct response.

#### Electrode implantation and surgery

Tetrodes were constructed from four twisted 13 µm coated nichrome wires (California Fine Wire). The electrode tips were gold plated to reduce electrode impedances to 300–800 kΩ at 1 kHz. Tetrodes were loaded into a custom-built microdrive containing 36 individually movable tetrodes (Lansink et al., 2007; Bos et al., 2017). In Rats 1 and 2, the drives targeted eight tetrodes to the perirhinal cortex (area 35/36; target coordinates in mm: −6.0 AP, 7.0 ML, 6.5 DV; Paxinos and Watson, 2013), eight tetrodes to dorsal hippocampal CA1 and CA3 area (−3.48 AP, 2.0 ML, 2.5 DV, aimed at the hippocampal pyramidal cell layer), eight tetrodes to secondary visual cortex (V2L; −6.0 AP, 5.8 ML, 2.8 DV), and eight tetrodes to the somatosensory cortex (S1 Barrel Field; −3.0 AP, 5.0 ML, 2.8 DV). For Rats 3 and 4, the drives were modified such that 13 tetrodes targeted the perirhinal cortex, 13 tetrodes the secondary visual cortex, and six tetrodes the somatosensory cortex, while maintaining the same target coordinates as in the previous drive.

Prior to surgery, rats received a subcutaneous injection of buprenorphine (Buprecare, 0.04 mg kg^−1^), meloxicam (Metacam, 2 mg kg^−1^) and enrofloxacin (Baytril, 5 mg kg^−1^). Anesthesia was induced by placing the animal in a closed plexiglass box filled with isoflurane vapor (3.0%). During surgery, the animals were mounted in a stereotaxic frame where anesthesia was maintained using isoflurane (1.0–2.0%) and body temperature was maintained between 35 and 36°C using a heating pad. Local anesthetic (lidocaine) was applied directly on the periosteum before exposing the skull. The skull was thoroughly cleaned with a 3% hydrogen peroxide solution to roughen the skull surface. The hydrogen peroxide was removed by rinsing the skull three times using saline. Six screws, from which one in the occipital bone served as ground, were inserted into the skull to improve the stability of the implant. After craniotomy and durotomy were performed, the drive was positioned over the craniotomies. The craniotomies were sealed using silicone adhesive (Kwik-Sil), the skull was covered with a layer of dental adhesive (OptiBond, Kerr) and primer (3M Transbond), and finally the drive was anchored using dental cement (Kemdent). Finally, all tetrodes were lowered into the superficial layers of the cortex. From the third day, tetrodes were gradually lowered toward their target location regions over a span of 7–10 d. Postoperative care included a subcutaneous injection of meloxicam once per day for 2 d following surgery and a single injection of enrofloxacin on the day following surgery.

#### Data acquisition

For data collection, the rat was connected to the recording equipment (Digital Lynx SX, 144 channels, NeuraLynx) via operational amplifiers (HS-36-LED, NeuraLynx) and a tether cable connected to a commutator (Saturn-5, NeuraLynx). During the days following surgery, tetrodes were lowered to their target location while their current location in the brain was estimated based on the distance the tetrode was moved in the drive and assessing the LFP and spike signals. During experiments, the neurophysiological signals were acquired continuously at 32 kHz, 0.1 Hz high-pass filtered, and stored on a hard drive for further processing. An infrared camera (EVI-D100P, Sony) positioned above the behavioral setup recorded behavioral activity illuminated by infrared LEDs at 25 frames per second. Per trial a high-speed video camera (M3, IDT) recorded whisker motion, illuminated by an infrared backlight (wavelength, 850 nm; Sygonix), at 500 frames per second. The training apparatus was controlled using a Field Programmable Gate Array and custom-written MATLAB software (MathWorks). Programmed commands and events recorded by sensors were sent to and recorded by the recording system as TTL pulses.

#### Histology

After the final recording session, currents (12 µA, 10 s) were applied to one lead of each tetrode to mark its endpoint with a small lesion. Twenty-four hours after lesioning, the animals were deeply anesthetized with Pentobarbital (Nembutal, Ceva Sante Animale, 60 mg ml^−1^, 1.0 ml intraperitoneal) and transcardially perfused with saline followed by a 4% paraformaldehyde solution, pH 7.4 (phosphate buffered). After postfixation, the brain was sectioned into 40–50-µm-thick slices using a vibratome. A Nissl staining (cresyl violet) was set to mark cell bodies. The sections were imaged and aligned to the 3D Waxholm reference atlas (Papp et al., 2014), using the QUINT workflow (Yates et al., 2019). Cells were excluded from analysis if their reconstructed recording locations were not within the anatomical borders of our regions of interest.

#### Spike sorting

Recorded spikes were detected and clustered offline using the Klusta package; spike clusters were manually curated using the Phy gui (Rossant et al., 2016). The quality of the curated clusters was assessed using SpikeInterface (Buccino et al., 2020). Single units were included for analysis if they met the following criteria: signal-to-noise ratio (of the mean waveform amplitude vs the background noise on the same channel) >2.5, <0.5% of spikes violating the refractory period, isolation distance >5, and firing rate over the whole session >0.1 Hz. Waveform characteristics were extracted using the SpikeInterface toolbox and used to classify cells as putative excitatory (pyramidal, PE) or putative inhibitory, fast spiking (FS) cells. First, cells were excluded from classification if no prominent spike waveform was detected (minimum required amplitude was 50 µV relative to background). Thereafter the distribution of the trough-to-peak latency was inspected, and a bimodal distribution, split at 400 µs, was found. Cells were then classified as PE if their trough-to-peak latency was larger than 400 µs or as FS if their trough-to-peak latency was smaller than 400 µs.

#### Multiple-comparisons correction

Reported results are corrected for multiple comparisons, using Bonferroni’s correction, except for the surrogate permutation tests (bootstrap estimation and permutation shuffling, see below), in which case an alternative to cluster-based correction was used.

#### Analysis of LFPs

For each session, one lead per recording area was selected for LFP analysis. LFP data recorded from CA3 in Rat 2 were inverted. First, these channels were included based on quality metrics: signal amplitude, signal standard deviations, and lack of 50 Hz artifact in the LFP, throughout recording sessions (Li et al., 2020). From the selected channels, for each session and each area, the channel with the strongest theta oscillation was selected based on manual curation, selecting for regular oscillations and the highest amplitude. The LFP data were visually inspected for noise artifacts, and trials with noise artifacts were excluded from the analysis. Finally, the leads were downsampled from 32 kHz to 1,000 Hz to improve processing speed using the SciPy decimate function.

#### LFP power and LFP–LFP coherence

We investigated the LFP dynamics by computing the time-frequency dependency of LFP power via the Morlet wavelet transform (7 cycles, computed using the MNE toolbox, tfr_array_morlet; Gramfort, 2013). For the 1/f correction of the power spectral density (PSD), the 1/f distribution was estimated using the IRASA method (Donoghue et al., 2020). To detect whether LFP amplitude was significantly increased and at which frequencies compared with baseline, we used Friedman's test for repeated measurements (*p* < 0.01) and a post hoc Wilcoxon signed-rank test (*p* < 0.01).

We focused on theta oscillations as we were interested in interareal communication between dHC and neocortex, and theta oscillations have been implicated in such communication during sensory and mnemonic operations (Buzsáki, 2009). To confirm this, we measured the LFP–LFP coherence between neocortical areas S1BF, V2L, and PER LFPs and dHC LFPs using the Weighted Phase Lag Index (WPLI; Vinck et al., 2011). This measure compensates for volume conduction and approaches zero in case of low coherence and 1 in case of high coherence. Statistical significance of the WPLI was evaluated using a permutation test shuffling the LFP phases (*n* = 500 shuffles).

#### Spike–LFP phase entrainment

For the spike phase analysis, the theta-band phase and power were estimated by first bandpass filtering the LFP [6–12 Hz, 4th order Butterworth filter, Elephant toolbox (Ulianych et al., 2021), butter], computing the analytic signal using the Hilbert transform (Elephant toolbox, hilbert) and finally taking its angle and magnitude to quantify the LFP phase and power, respectively. While the LFP was subsampled at 1,000 Hz and the spike data at 32 kHz, we interpolated the LFP phase data onto the spikes to retrieve the spike-triggered phase distribution. When computing spike–LFP coherence for a given cell, we ensured to select an LFP signal from a different tetrode than the tetrode on which that cell was recorded.

First, we asked what fraction of each cell population was significantly entrained during subsequent behavioral epochs (Fig. 5). Cells were considered significantly entrained if their spike-triggered phase distributions were nonuniform, as measured using the Rayleigh's test for uniformity (Astropy toolbox; The Astropy Collaboration et al., 2022; stats.rayleightest; with *p* value lower than *α* = 0.05). The same measure was used to detect whether cells were phase entrained by same-area LFP or dHC LFP. The significance of the difference between the fractions of cells locking was assessed using a bootstrap approach: the fraction of cells locking was recomputed by resampling from the cell population with replacement (1,000 resamples); based on the reshuffled data, the 95% confidence intervals were constructed. Fractions of cells were considered significantly different if the confidence intervals did not overlap. To compare the strength of phase entrainment on same-area LFP and dHC, we used the pairwise-phase consistency (Vinck et al., 2012); the significance of the difference between these types of LFP signal was assessed with Wilcoxon's signed-rank test.

#### Correlation between firing activity and theta power

To investigate the modulation of firing activity by theta power, we grouped the spiking data in deciles based on theta power derived from same-area LFP: per trial 1 s segments of theta-band filtered LFP power were sampled from the period −2 to 10 s around trial start. Over the full session, these segments were ranked in deciles based on their power. For the same segments, we computed the average firing rate for each recorded cell. We visualized the cell's firing rate versus theta power (Fig. 6). Then we determined whether a cell was significantly modulated by theta power by comparing the bootstrap estimated average firing rate in the first decile versus the bootstrap estimated average in the last decile (bootstrap resampling from firing rates per 1 s segment, 1,000 resamples, 95% confidence intervals). Cells were considered significantly modulated (i.e., their firing rate increases or decreases as a function of theta power) when the confidence intervals from the estimated averages did not overlap.

#### Temporal dynamics of phase entrainment

Next, we investigated the temporal dynamics of phase entrainment (Figs. 7, 8). We computed the cell's phase-locking strength over LFP phases in all trials, binned using a 200 ms wide window shifted by 10 ms steps over a −0.5 to +1.5 s interval relative to sample start. We chose this time window as it included three epochs of interest: object approach, discrimination, and reward site approach. As the behavior was self-paced, the duration of each trial phase varied between trials, and taking a longer time window would introduce more variance in the behavior the further away it is measured from the aligning trigger (sample onset). Because of the narrow window (200 ms), and the resulting low spike counts, we used the pairwise-phase consistency (PPC; Vinck et al., 2012) to evaluate the locking strength, as the PPC is not biased by low sample size. We used permutation tests (*n* = 500 samples) to assess the significance of the measured PPC in each time bin. For each time bin, the PPC was computed on *Nspikes* LFP phases which were randomly sampled out of all LFP phases recorded during a session in the −0.5 to +1.5 s time window around sample start, where *Nspikes* is the number of spikes in the bin under scrutiny. The *p* value was measured as the empirical datapoint's rank in the shuffled distribution divided by the number of permutations (i.e., if 2 out of 500 shuffled datapoints were higher than the empirical data point, then *p* < 0.01). This method corrects for the nonuniformity of the LFP phase distribution (hippocampal theta oscillations may take the form of a “sawtooth” pattern) and the number of spikes occurring during a bin. To further reduce noise in this analysis, cells were excluded if, during any of the behavioral epochs, <50 spikes were measured throughout the full recording session. Cells were deemed significantly phase entrained if *p* < 0.01 but were excluded from being significant if bins were not marked as significant for at least 100 consecutive milliseconds ( = 10 consecutive bins) during the full analyzed time window of 2 s duration. This approach was derived from cluster correction, a multiple-comparisons correction for permutation testing over many samples (Maris and Oostenveld, 2007). We visualized the timing of phase entrainment over the recorded population by normalizing the PPC per cell to its maximum value (compare Figs. 7*B*, 8*A4–B3*).

To report phase entrainment across all behavioral epochs (the outcome and baseline epochs were not included in the −0.5 to +1.5 s time window), we also report the phase entrainment on data aligned to the start of each behavioral epoch (baseline, object approach, discrimination, reward site approach, and reward). This approach corrects for the variance introduced into the data by the self-paced free behavior of the animal (i.e., variance in sampling duration, locomotion, etc.). Significance between the PPC values measured in these behavioral epochs was assessed using a pairwise *t* test (*p* < 0.05; Bonferroni’s corrected).

#### Modality selectivity of phase entrainment

We assessed whether cells were selectively phase entrained during tactile, visual, and/or multimodal stimulus conditions. To this end, we computed the PPC during the sensory discrimination epoch per modality. Spike-triggered phases were grouped by the modality of the presented trials. A threshold for the significance of a cell's phase entrainment by a modality was determined using random permutation testing: the PPC was measured 500 times on random LFP phases drawn without repetition from the sampling epochs. The *p* value was measured as the rank of the empirical PPC in the shuffled distribution (i.e., rank = 2 out of 500: *p* = 0.04). Measured PPC values were considered significant if *p* < 0.05.

To verify the significance of the reported fractions of cells phase entrained per modality (Fig. 9*A1–C1*), the fractions were re-estimated using a bootstrap procedure (1,000 shuffles) and 99% confidence intervals of the fraction of phase-entrained cells were estimated. Reported fractions were considered significant if the estimated confidence intervals did not overlap with zero.

The average strength of phase entrainment per recording area (Fig. 9*A2–C2*) was computed over all cells previously marked as phase entrained. Differences between modality conditions were evaluated with the Kruskal–Wallis test and a post hoc Wilcoxon's signed-rank test (*p* < 0.01).

Finally, we assessed whether modulations in firing rate or theta amplitude could explain any modality-selective effects. To determine the effect of variable firing rate between modalities, we recomputed the PPC for each cell and each modality but sampling only *Nspikes* for each modality, where *Nspikes* is the number of spikes recorded in the modality with the fewest spikes. Thus any effect purely driven by an increase or decrease in firing rate should not affect the outcome of this analysis. To determine the effect of theta amplitude, we measured the theta amplitude (i.e., the power from theta-bandpassed LFP) occurring during spikes in every modality condition. Differences between groups were assessed using the Kruskal–Wallis test and considered insignificant if *p* > 0.01.

## Results

### Electrophysiological recordings in a multisensory object discrimination task

Rats were trained on a two-alternative forced choice discrimination task with solid, 3D objects (Fig. 1*A–C*, c.f. Fiorilli et al., 2024). The rat's behavior was self-paced and tracked using infrared (IR) light sensors in the object sampling area (Fig. 1*A*, IR sensor 1) and the reward sites (Fig. 1*A*, IR sensors 2 and 3). Following trial start, the door blocking the sampling area was lowered and the rat could detect the stimulus (Fig. 1*A*, sampling area). In each trial, one of two objects (Fig. 1*C*, object A or object B) was presented. The sensory modality wherein the object was detectable during a given trial varied between tactile (T, object reachable for whiskers but in darkness), visual (V, object not reachable for whiskers but illuminated), or both senses combined (multisensory; M, object reachable for whiskers and illuminated). In order to sample the object, the rat reached with his head into the sampling area (triggering IR 1, sample start). Following object sampling, the rat retracted its head from the sampling area and moved to one of the arms of the elevated platform to retrieve a reward (triggering IR 2 or 3, reward poke). A sucrose solution reward was delivered when the rat poked at the reward site associated with the object on display. Following a poke, an ITI of 12 s started. In case of an incorrect response, no sucrose was delivered, and the ITI was extended by 8 s. Because the experiment was self-paced, the time spent in each trial phase varied per animal and trial (see Materials and Methods).

**Figure 2.**
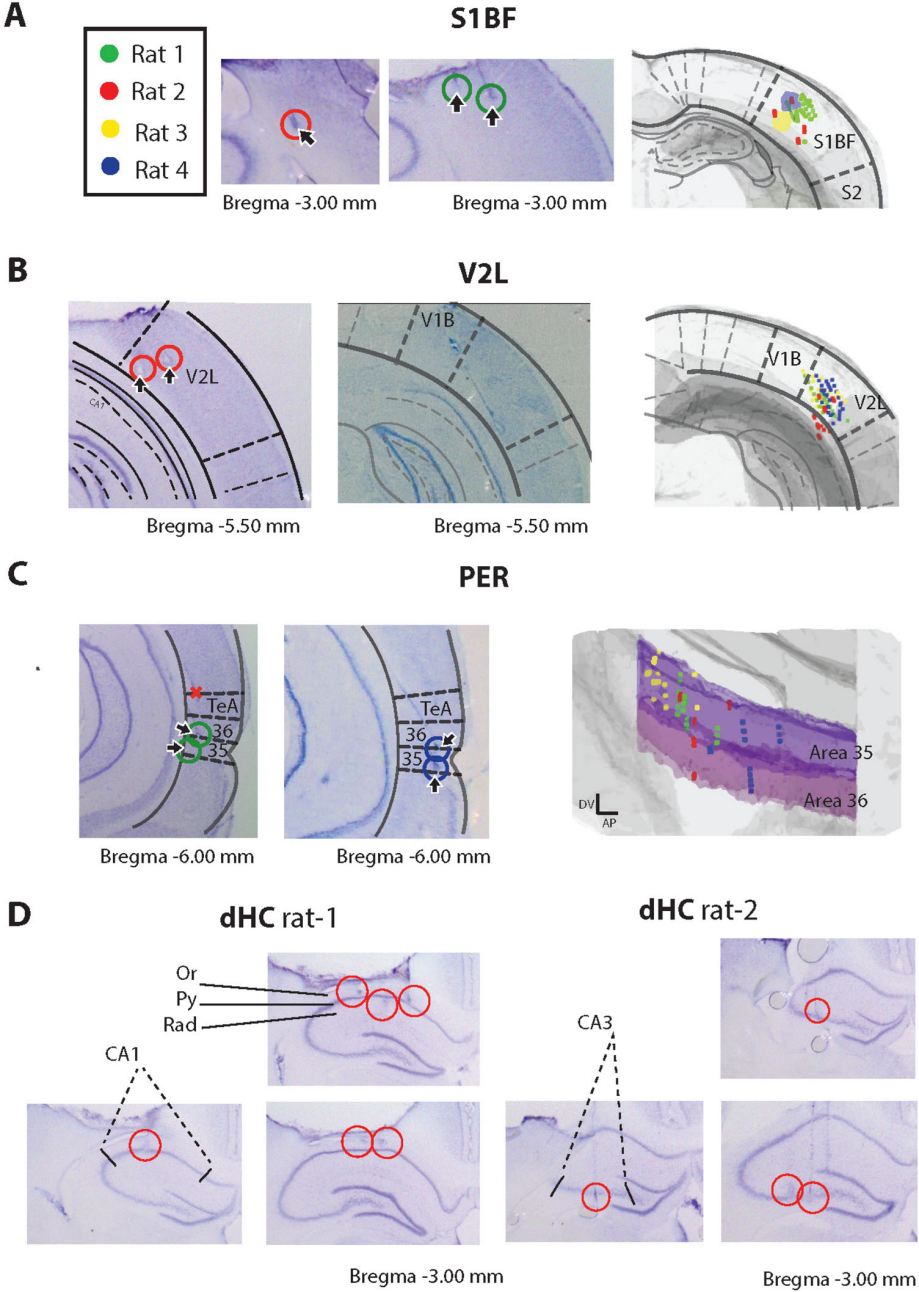
Histological verification of recording sites. Histological sections were aligned to the 3D Waxholm atlas (Papp et al., 2014). Following the alignment of the histology sections, the tetrode endpoints were tracked and registered to an area. Tetrodes which were tracked outside the target areas were excluded. ***A***, Data are shown for all four animals in area S1BF; ***B***, data recorded from four animals in V2L; and ***C***, data recorded from four animals in PER. ***D***, Examples of recording sites in dHC in Rat 1 CA1 (left) and Rat 2 CA3 (right).

We segmented the behavioral trials into five 500 ms epochs, using the infrared sensors to track the animal's behavior (Fig. 1*B*): object approach (the animal navigated to the sampling area, 0.5–0 s before sample start), sensory discrimination (the animal entered the sampling area and identified the object, 0–0.5 s following sample start), reward site approach (the animal moved from the sampling area and navigated to its site of choice, either the left or right reward site, −1 to −0.5 s before reward poke), outcome (the animal poked the reward site and was rewarded upon a correct choice, 0–0.5 s following reward poke), and baseline (taken as the interval of 6–6.5 s following the reward poke). Trials wherein the animal started withdrawing from the sampling area before 200 ms were excluded from analysis. Rats (*n* = 4) learned to discriminate between the two objects and reported the correct object identity more frequently in the multisensory condition *M* than those in the unisensory conditions (Fig. 1*E*; *T* vs *M*: *t*_(24)_ = 6.30, *p* < 0.01, paired *t* test and *V* vs *M*: *t*_(24)_ = 7.03, *p* < 0.01, paired *t* test), averaged over four animals, 25 sessions: *T*, 72%; *V*, 72%; *M*, 84% correct response rate (see Table 1 for trial count per animal and modality), while performance between unisensory modalities did not differ significantly (*T* vs *V*, *t*_(24)_ = −0.85; *p* = 0.40; paired *t* test). For all modalities, performance was significantly higher than during probe trials (51% correct response rate during probe trials; chance level, 50%; *T* vs *P*: *t*_(20)_ = 3.87, *p* < 0.01; *V* vs *P*: *t*_(20)_ = 3.75, *p* < 0.01; *M* vs *P*: *t*_(20)_ = 6.81, *p* < 0.01, paired *t* test). Behavioral performance was stable across recording days (example data in Fig. 1*D*). Ensemble activity and LFP traces were recorded from the somatosensory barrel cortex (S1BF), secondary visual cortex (V2L), perirhinal cortex (PER), and dorsal hippocampus (dHC, subfields CA1 and CA3). The recording sites were verified using histological reconstruction of tetrode tracks (Fig. 2; see Materials and Methods). Cells were only included if their reconstructed recording locations were within the region of interest.

**Table 1.** Number of trials recorded per modality, per animal

	Rat 1	Rat 2	Rat 3	Rat 4	Total
*T*	233	466	319	338	1,356
*V*	200	572	239	206	1,217
*M*	177	415	127	61	780

### Theta oscillations in the hippocampus and neocortex during locomotion and sensory discrimination

**Figure 3.**
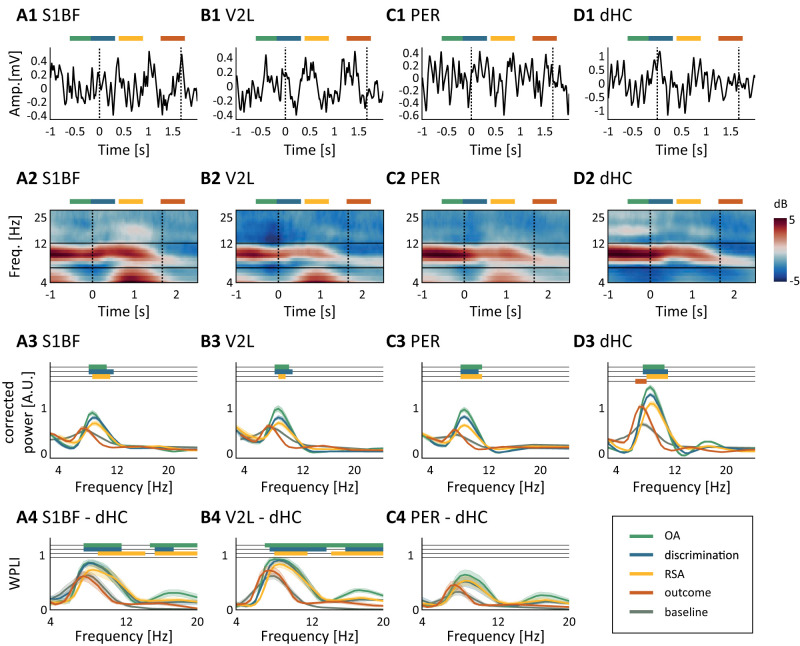
Hippocampal theta power is modulated by task engagement and cortical theta oscillations cohere with hippocampal theta rhythm. ***A1***, Example S1BF LFP trace (1–25 Hz). The dashed black vertical lines mark average trigger times for sample start and nose poke for reward. On top of the trace, the epochs for segmentation are marked [green, OA (object approach); blue, sensory discrimination; yellow, RSA (reward site approach); orange, outcome]. ***A2***, Time-frequency plots of baseline-corrected power (color code, power corrected for baseline in decibels) in the low-frequency domain (*y*-axis, 4–25 Hz) aligned to sample start. Theta oscillations are present before *t* = 0, that is, during locomotor approach toward the sampling area and diminish following the onset of the outcome epoch, likely correlated to immobility. ***A3***, 1/f corrected PSDs per behavioral epoch (epochs colored as in ***A1***). Colored horizontal bars mark frequencies with a significant power difference compared with baseline (*p* < 0.01; Friedman test; post hoc one-sided Wilcoxon signed-rank). ***A4***, Mean debiased WPLI ± SEM measured between dHC and areas S1BF, V2L, PER, per behavioral epoch (same color code as ***A1***,***A2***). The WPLI was significant during all behavioral epochs (*p* < 0.05; permutation test; not annotated), and an increase of WPLI with respect to the baseline was measured in S1BF and V2L (horizontal color bars, *p* < 0.01; Friedman test; post hoc one-side Wilcoxon signed-rank). ***B–D***, Same as ***A*** but for areas V2L, PER, and dHC, respectively.

Hippocampal theta oscillations (6–12 Hz) have been shown to increase in power during particular behaviors such as locomotion and whisking (Grion et al., 2016; Kropff et al., 2021). We therefore expected increased HPC theta LFP power during engagement of the rat in our behavioral paradigm. For each session, we selected one lead per recorded area per session (for example data, see Fig. 3*A1–D1*, areas S1BF, V2L, PER, dHC; see Materials and Methods) and computed the average LFP power in the theta range across the lead [Fig. 3*A2–D2*; data pooled across all sessions: S1BF (all four rats); 21 sessions, V2L (all four rats); 23 sessions, PER (all four rats); 21 sessions, dHC (two out of four rats); 17 sessions]. A clear peak in the theta range was visible in all recorded areas before and during epochs of object approach, sensory discrimination, and reward site approach, which decreased following the nose poke for reward (Fig. 3*A2–D2*). To quantify the modulation of theta power during behavior, we computed the PSD per behavioral epoch, corrected it for the 1/f distribution, and compared it against baseline (Fig. 3*A3–D3*). Relative to baseline, this corrected measure of theta power was significantly enhanced during the object approach, sensory discrimination, and reward site approach epochs in all areas (*p* < 0.01 for all areas; one-sided Wilcoxon signed-rank test). Theta power was also significantly increased during the outcome epoch in dHC, but not in the neocortical areas (*p* < 0.01; one-sided Wilcoxon signed-rank test; multiple comparison corrected).

The stimulus onset was paired with a shift in the theta peak frequency in areas S1BF, V2L, and dHC (Fig. 3*A2–D2*,*A3–D3*; nonoverlapping bootstrapped 99% confidence intervals). The onset of the frequency shift nearly coincided with the onset of stimulus and reached its maximum ∼500 ms following stimulus onset. Theta frequency has been shown to depend on acceleration in body motion (Kropff et al., 2021). This likely explains the shift detected here, as the animals slow down their movement when approaching the object and afterward accelerate again.

### Neocortical theta oscillations cohere with the hippocampal theta rhythm

The LFP recordings in neocortical areas S1BF, V2L, and PER showed similar temporal dynamics as dHC in the theta range (Fig. 3*A3–D3*). These theta oscillations could be generated locally, within the neocortical areas. On the other hand, previous research has suggested that theta oscillations recorded from rat neocortex may arise through volume conduction of the LFP from the dHC (Gerbrandt et al., 1978; Sirota et al., 2008; Vinck et al., 2016). Thus the neocortical theta oscillations could be a reflection of theta oscillations generated in dHC.

We quantified the phase coherency between dHC and neocortical areas using the WPLI (Vinck et al., 2011), a metric of phase synchronization that is less affected by volume conduction, computed between dHC and neocortical LFP theta oscillations (Fig. 3*A4–C4*). We found significant phase coherence during all behavioral epochs between theta oscillations recorded from all three areas of the neocortex (*p* < 0.05; permutation test) and those recorded from dHC. As shown in Figure 3*A4–C4*, the strength of phase synchronization (WPLI) differed per behavioral epoch, with the strongest WPLI values being reached during object approach, sensory discrimination, and reward site approach phases (*p* < 0.01, one-sided Wilcoxon signed-rank test). All three neocortical areas showed similar profiles with their LFP signals referenced to dHC, and no significant difference between areas was found (*p* > 0.01; Friedman test), even though only S1BF and V2L reached a significant increase of WPLI coherence compared with baseline during several epochs. Taken together, these results show a widespread presence of coherent theta oscillations throughout the hippocampus and neocortex during task engagement by the animal.

### Phase entrainment of neocortical and hippocampal cells by theta oscillations

**Figure 4.**
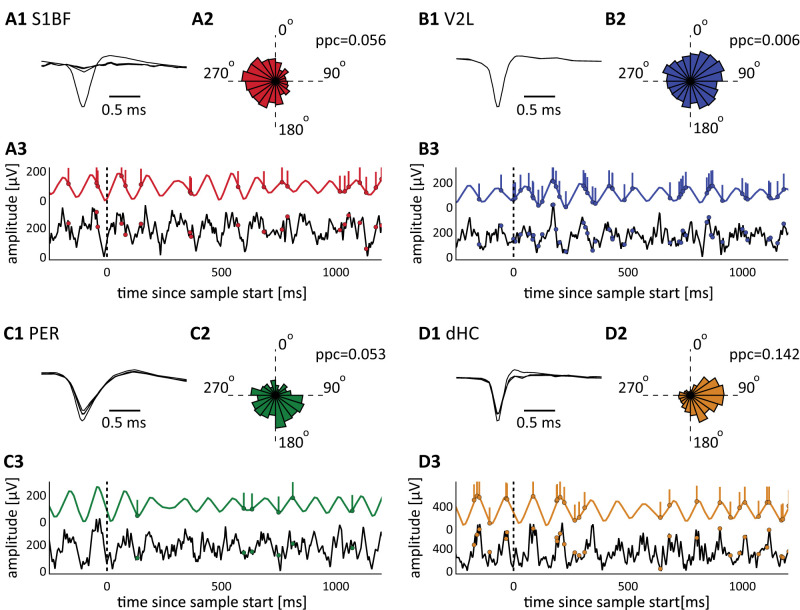
Example data displaying phase entrainment to theta oscillations in simultaneously recorded activity across four areas. The data in this figure were recorded simultaneously and display four cells significantly phase entrained to theta oscillations on same-area LFP. ***A1***, Average spike waveforms for an S1BF cell on four tetrode leads. ***A2***, Theta phase distribution (in degrees) of spikes of the same S1BF cell recorded during a full session (bin width is 18°). ***A3***, A single trial example of broadband (4–120 Hz, bottom, black) S1BF LFP plus theta filtered (6–12 Hz, red, top) S1BF LFP and the single-unit firing of the S1BF example cell following the onset of sensory discrimination (vertical black dotted line; *t* = 0). Colored vertical lines: spike times for the example units. ***B–D***, Same as ***A*** but for areas V2L, PER, and dHC, respectively.

We next investigated the phase entrainment of cells along the corticohippocampal hierarchy (areas S1BF, V2L, PER and dHC) during the different behavioral epochs, as we expected cells in these areas to be phase entrained depending on the engagement of these areas during specific stimuli and behaviors. We analyzed spiking activity from single units recorded across the four areas (example data in Fig. 4; S1BF, 167 cells; V2L, 371 cells; PER, 239 cells; dHC, 220 cells) while relating this activity to same-area and dHC theta (6–12 Hz) oscillations. We will refer to phase entrainment as the biasing of a cell's activity toward a specific phase in LFP activity as a result of external input, in the case of S1BF and V2L cells: incoming sensory information or top-down input conveyed through a descending pathway (Kleinfeld et al., 2016). The statistical significance of theta phase entrainment of individual cells was determined using Rayleigh's test (cells are marked as phase entrained if *p* < 0.05). Phase entrainment was quantified during each behavioral epoch and during the sampling epoch separately for each modality condition. In the following sections, we consider a cell phase entrained during the discrimination epoch if it is entrained during at least one of the three presented modality conditions (we further expand on modality selectivity in the final paragraph of the Results).

**Figure 5.**
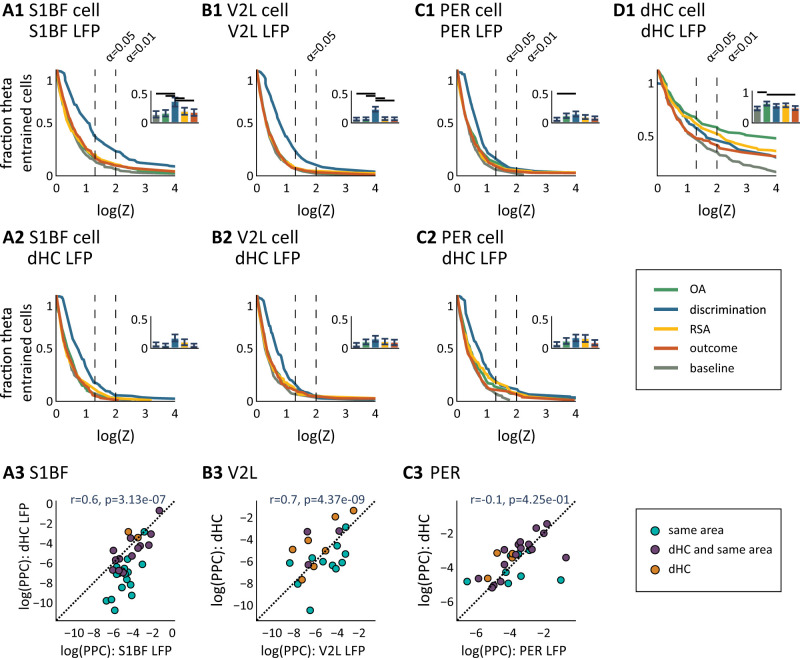
Phase entrainment of neural populations in areas S1BF, V2L, PER, and dHC. Percentage of cells locking onto theta depends on behavioral epoch and reference area. ***A1***, S1BF cell phase entrainment on same-area (=S1BF) LFP. This plot shows the fraction of cells (*y*-axis) with a *p* value (determined by Rayleigh's test) lower than the given log(Z) value on the *x*-axis, measured per behavioral epoch [gray, baseline; cyan, OA (object approach); blue, sensory discrimination; yellow, RSA (reward site approach); orange, outcome]. For instance, a log(Z) of 2 means that the spike–LFP locking was significant at *p* < 0.01 for the given fraction of cells. The vertical dashed lines mark the alpha levels (from left to right: 0.05, 0.01). The inset shows the fraction of entrained cells at *α* = 0.05, error bars show 95% bootstrapped confidence intervals and black horizontal bars mark significantly different pairs. ***A2***, Same as ***A1***, but now S1BF cell phase entrainment is measured against dHC LFP. ***A3***, Phase-entrainment strength (PPC) measured on same-area (i.e., S1BF, *x*-axis) theta versus phase-entrainment strength measured on dHC LFP (*y*-axis) are correlated (Pearson’s *r*). Using Rayleigh's test (*p* < 0.05) cells are labeled as locking on same area only (cyan), dHC only (orange), or both same area and dHC (purple). ***B–D***, Same as ***A*** but for areas V2L, PER, and dHC, respectively.

**Figure 6.**
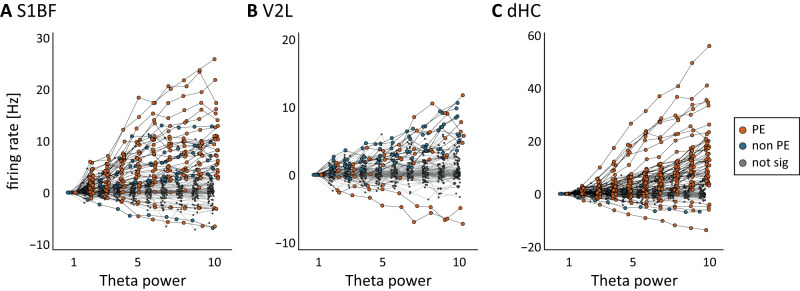
Firing rate modulation of cells by theta power. The firing rate of phase-entrained cells is more likely to be modulated by increasing theta power than that of nonphase-entrained cells. For areas (***A***) S1BF, (***B***) V2L, and (***C***) dHC, theta power was measured in 1 s segments and grouped in deciles (*x*-axis). For each recorded cell the firing rate per decile was measured (see Materials and Methods). Firing rate was normalized relative to firing rate in the first decile. Data are color coded; orange, phase-entrained cells significantly modulated by theta power; blue, nonphase-entrained cells significantly modulated by theta power; gray, cells not significantly modulated by theta power.

**Figure 7.**
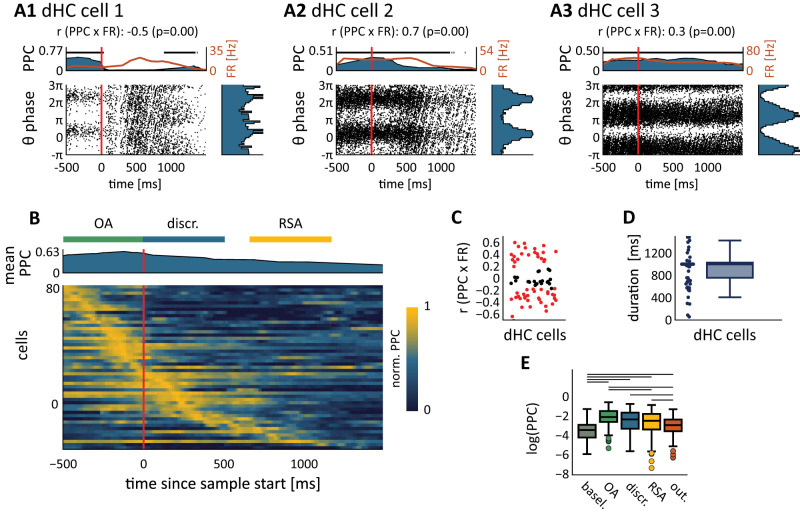
Phase-entrainment dynamics of dorsal hippocampal cells. ***A***, Three examples of dHC cells displaying various temporal phase-entrainment dynamics with respect to same-area LFP. Bottom left, Spike timing (*x*-axis) compared with LFP theta phase (*y*-axis), aligned to sensory discrimination onset; spikes were sampled over all trials from a full session. The oblique striped patterns are bursts of cell firing. The PPC corrects for a bias due to bursts by measuring phase consistency between trials. Top, PPC (black curve) and firing rate (FR, in Hz); red curve, right *y*-axis) computed over 200 ms bins using a sliding step of 10 ms. Horizontal black bars and blue shaded areas denote bins with significance phase entrainment (*p* < 0.01, permutation testing). Right, Phase histogram for spikes emitted during significant bins (as marked in the top plot). ***B***, Overview of phase entrainment in the dHC population. Only cells significantly phase entrained, as per this analysis, were included (*n* = 80, sampled across 17 sessions). The dHC population shows phase entrainment spread throughout the trial. Cells have been ordered according to the time bins at which they reached their peak in normalized PPC value (see Materials and Methods). Heatmap, for each cell (*y*-axis) the normalized PPC was computed over time as in ***A*** (*x*-axis). Top, Mean of the normalized PPC for the 80 recorded cells. Behavioral epochs relative to the average onsets are marked on top (OA, object approach; discr., sensory discrimination; RSA, reward site approach). ***C***, Correlation strength between a cell's firing rate (FR) and phase entrainment (PPC), cells with significant correlations are marked red (*p* < 0.01; Pearson’s correlation). ***D***, Box plot and its underlying distribution of locking duration for dHC cells measured using the PPC (see Materials and Methods). ***E***, PPC computed per behavioral epoch, black bars denote significant differences between pairs of behavioral epochs (*p* < 0.01; Wilcoxon's signed-rank test).

**Figure 8.**
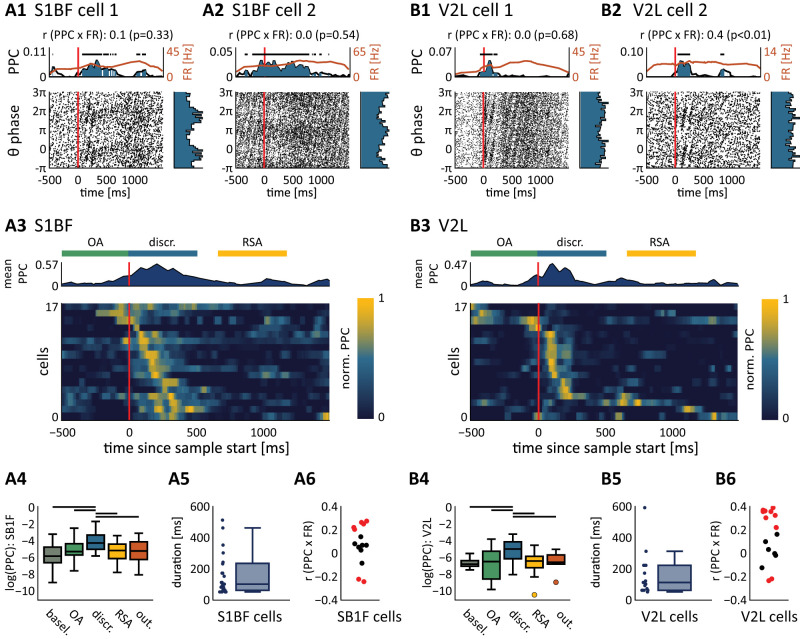
Phase entrainment of somatosensory and visual cortex populations. Same as Figure 7, but now for the S1BF population (***A***, left) and V2L population (***B***, right). Phase entrainment is measured on LFP recorded from the sensory areas (same-area LFP). ***A1***, ***A2***, Spike-triggered phases for two example cells aligned to sample start. ***A3***, Heatmap, normalized PPC for all phase-entrained S1BF cells (*n* = 17, recorded in 12 sessions), aligned to sample start. Cells show transient phase entrainment following stimulus onset and rarely phase locked in subsequent trial epochs. Above the heatmap the mean normalized PPC is visualized, including bars denoting the object approach (OA), sensory discrimination (discr.), and reward site approach (RSA) epochs. The transient nature of phase locking is further emphasized by segmenting the data into behavioral epochs. ***A4***, PPC per behavioral epoch (epoch abbreviations as in ***A3***; out., outcome). Black bar denotes significant difference between pairs of behavioral epochs (*p* < 0.01; Wilcoxon signed-rank test). ***A5***, S1BF cells are phase entrained only in short time windows, as reported by the duration of significant phase locking (*y*-axis, boxplots). ***A6***, Pearson’s correlation between firing rate (FR) and PPC (computed over −0.5 s to +1.5 s relative to sample start; Fig. 7*C*). ***B***, Same as ***A*** but for area V2L (*n* = 17 cells, recorded in 12 sessions).

**Figure 9.**
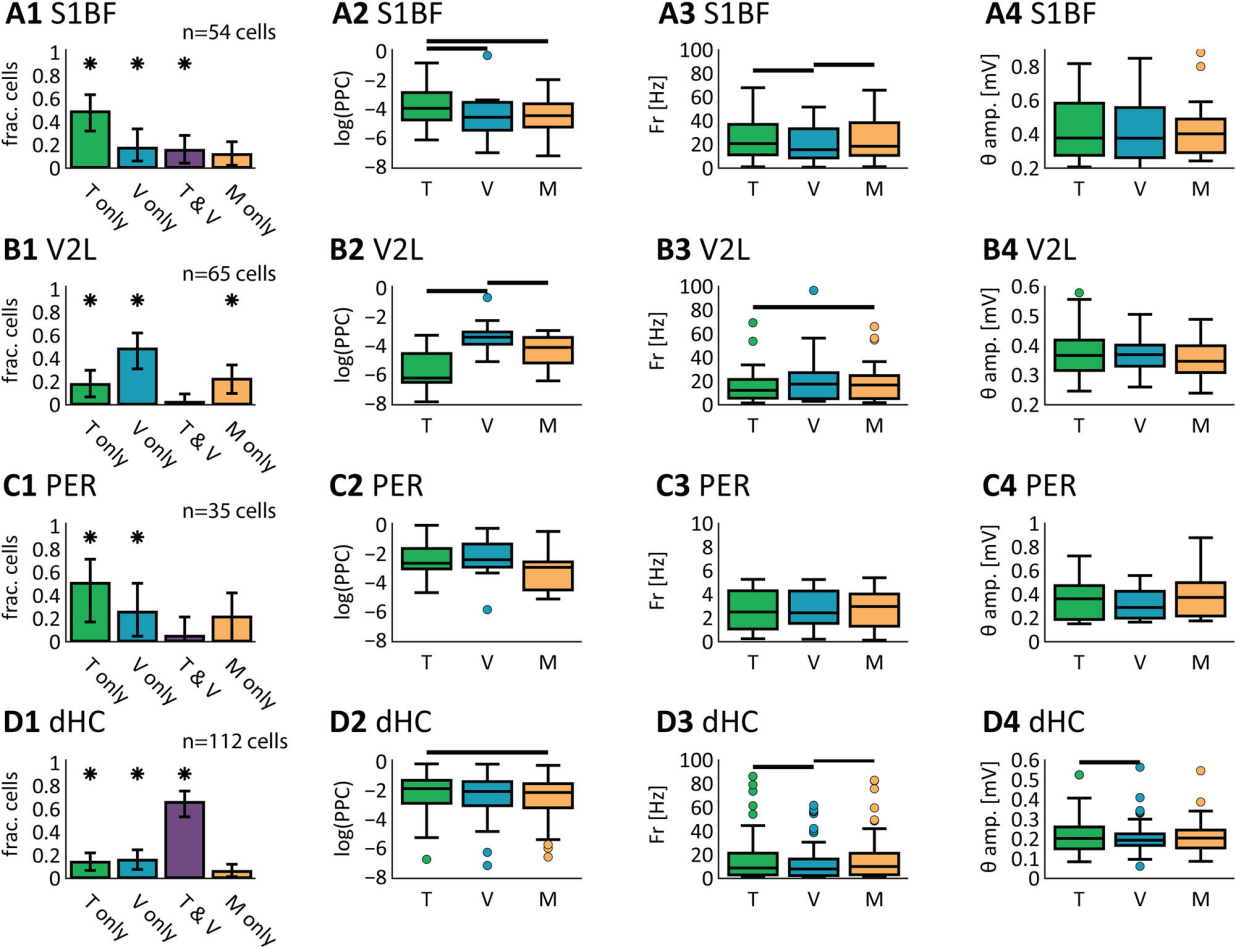
Phase-locking consistency depends on sensory modality. The phase-entrainment of cells was assessed per stimulus modality. ***A1***, Cell phase-entrainment selectivity for modality condition. All cells significantly phase entrained (PE; random permutation testing; *p* < 0.01) during the discrimination epoch were classified as selective for tactile-only (green, *T*-only; PE during *T* trials), visual-only (blue, *V*-only; PE during *V* trials), tactile–visual (purple, *T* & *V*; PE during *T* and *V* trials), and multimodal-only (orange, *M* only; PE during *M* trials, but not during *T* and *V* trials). Per category, the fraction of the total cell count is displayed (*y*-axis); cells were excluded if they were not PE during tactile-only, visual-only, and multimodal-only (S1BF, 29 cells; V2L, 23 cells; dHC, 95 cells included). Error bars denote 99% bootstrapped confidence intervals (1,000 shuffles), significant fractions are marked by an asterisk (a fraction is considered significant if the 99% CI is above zero). ***A2–A4***, Further analyses show that the modality selectivity of phase entrainment (***A2***) is not purely driven by fluctuations in firing rate (***A3***) or fluctuations in LFP theta-band amplitude (***A4***). For these control analyses, the cells selectively phase entrained on their preferred modality are included (see ***A1***; S1BF, *T*-only; V2L, *V*-only; dHC, *T* & *V*). ***A2***, The strength of phase entrainment for modality-selective cells (*y*-axis, Log of PPC) during *T* (green), *V* (blue), and *M* (orange) conditions. Horizontal black bars denote significant differences (Kruskal–Wallis; post hoc Wilcoxon signed-rank; *p* < 0.01). ***A3***, To test for biases in firing rate during the *T*, *V*, and *M* condition, we compared the firing rate between conditions and found no significant differences (Kruskal–Wallis; *p* > 0.01). ***A4***, Same as ***A3***, but now comparing theta oscillation amplitude between modal conditions; no significant difference was found (Kruskal–Wallis; *p* > 0.01). ***B–D***, Same as ***A*** but for areas V2L, PER, and dHC, respectively.

In both sensory neocortical areas, we found the largest fraction of significantly entrained cells on same-area LFP during the sensory discrimination epoch (Fig. 5*A1–D1* and Table 2; S1BF, 37% (29–43%) of cells phase entrained, mean plus 95% confidence intervals; V2L, 23% (18–27%) of cells phase entrained), while the fraction of entrained cells was significantly lower during the other four epochs (bootstrap test, 95% CI). Interestingly, a higher fraction of cells was detected as phase entrained for S1BF and V2L populations to same-area LFP compared with hippocampal LFP (bootstrap test, 95% CI; compare Fig. 5*A2–B2*, Table 3). Thus we asked whether, if cells were phase entrained by same-area LFP, they were also phase entrained by dHC LFP. To answer this question, we labeled cells as phase entrained by same-area LFP only, by dHC-only, or by both, using Rayleigh's test. Only a small fraction of phase-entrained S1BF and V2L cells were significantly locking on both dHC and same-area LFP, while the largest fraction of cells was phase entrained on same-area LFP only (S1BF, 58% of cells only on same-area LFP, 12% of cells only on dHC LFP, 31% of cells on both same-area LFP and dHC LFP; V2L, 52% of cells on same-area LFP, 31% of cells on dHC LFP, 17% of cells on same-area and dHC LFP). We validated these findings by comparing them against another, unbiased, measure of spike–LFP locking: the PPC (Vinck et al., 2012). We measured the PPC on same-area and dHC LFP during sensory discrimination for each cell that significantly locked on either LFP (Fig. 5*A3–B3*). The PPC was higher when measured on same-area LFP compared with dHC LFP for S1BF cells (*T* = 338; *p* < 0.01; Wilcoxon's signed-rank test) but not for V2L cells (*T* = 162; *p* = 0.10; Wilcoxon's signed-rank test), indicating that the entrainment of S1BF cells is stronger with same-area LFP than dHC LFP but, on a population level, the entrainment of V2L cells is not preferentially stronger on same-area LFP compared with dHC LFP.

**Table 2.** Percentage of cells locking on same-area LFP per behavioral epoch (mean, bootstrap 95% confidence interval; OA, object approach; RSA, reward site approach)

	Baseline	OA	Sampling	RSA	Reward
S1BF	14 (8–19)	16 (10–21)	37 (29–43)	20 (14–26)	18 (12–24)
V2L	6 (4–9)	7 (4–9)	23 (18–27)	7 (5–10)	6 (4–9)
PER	5 (3–8)	12 (7–16)	15 (11–18)	9 (6–13)	8 (5–11)
HPC	47 (39–53)	63 (55–69)	55 (48–61)	58 (51–64)	48 (40–54)

Fractions are considered significantly different if bootstrapped confidence intervals do not overlap.

**Table 3.** Percentage of cells locking on dHC LFP per behavioral epoch (mean, bootstrap 95% confidence interval; OA, object approach; RSA, reward site approach)

	Baseline	OA	Sampling	RSA	Reward
S1BF	6 (2–10)	4 (1–8)	17 (11–23)	10 (5–16)	4 (1–7)
V2L	5 (2–9)	10 (6–15)	16 (10–21)	11 (6–15)	10 (6–15)
PER	6 (3–10)	12 (7–17)	18 (11–24)	16 (10–23)	9 (5–14)
HPC	47 (39–53)	63 (55–69)	55 (48–61)	58 (51–64)	48 (40–54)

Fractions are considered significantly different if bootstrapped confidence intervals do not overlap.

A small fraction of PER cells was phase entrained on same-area theta LFP during the sampling epoch [15% (11–18%) of cells phase entrained]. Cell preference for same-area or dHC LFP was heterogeneous (30% PER-LFP only, 19% of cells on dHC LFP, and 51% on both same-area LFP and dHC LFP). The PER cell population tended to be somewhat more strongly entrained to dHC LFP than same-area LFP (Fig. 5*C3*; *p* = 0.02; Wilcoxon's signed-rank test). Thus, we did find a rather sparse phase entrainment in PER, which did occur preferentially with respect to dHC LFP.

Given its role in episodic and spatial memory, the hippocampus is likely involved in at least some of the behaviors required to perform the task (e.g., object discrimination and recognition, spatial choice, and navigation). Indeed, we found a large fraction of cells phase entrained in dHC during all behavioral epochs (with a minimum of 47% during baseline and a maximum of 63% during object approach; Fig. 5*D1*). We recorded cells from dorsal hippocampal subfields CA1 and CA3, the pyramidal cells of which show functional differences in firing rate and phase entrainment (Mizuseki et al., 2012). These previous findings prompted us to investigate potential differences between phase entrainment in CA1 and CA3 in our task. We split the dHC cell population by subfields CA1 and CA3 and did not find differences in the fraction of phase-entrained cells (data not shown; bootstrap test, 95% CI), although the phase entrainment of CA3 cells was stronger than CA1 cells during sensory discrimination (*T* = 768; *p* < 0.01; Wilcoxon's signed-rank test) and reward site approach epochs (*T* = 942; *p* < 0.01; Wilcoxon signed-rank test). In the subsequent analyses, we pooled CA1 and CA3 data under a common denominator (dHC) because the fractions of phase-entrained cells in both subregions were not statistically different, and no further major differences were found between these subregions.

Next, we aimed to gain insight into how same-area theta power and neural activity are related to each other. To do so, we grouped the theta power of same-area LFP in 1 s epochs and grouped them in deciles. For every cell, we then computed the average firing rate per decile. Cells were considered significantly modulated if their firing rate was higher or lower than their firing rate in the first decile (bootstrap test, 1,000 resamples, 95% confidence intervals; see Fig. 6 and Materials and Methods). Further, we distinguished between phase-entrained cells and nonphase-entrained cells, to study whether or not phase-entrained cells are more likely modulated by theta power than nonphase-entrained cells.

In areas S1BF and dHC, we found a significant increase in average firing rate for phase-entrained cells [average change in firing rate: S1BF: +3.38 Hz (2.38–4.59 Hz); dHC: +4.55 Hz (3.39–6.09 Hz), mean plus 95% bootstrap confidence intervals] but not for nonphase-entrained cells [S1BF: 1.14 Hz (0.47–2.01 Hz) Hz; dHC: −0.09 Hz (−0.67 to 0.49 Hz)]. In V2L and PER, an increase in theta power was not paired with an increase in firing rate, neither for phase-entrained cells [V2L: 0.86 Hz (0.40–1.46 Hz); PER: 0.12 Hz (−0.10 to 0.38 Hz)] nor nonphase-entrained cells (V2L: 0.83 Hz (0.59–1.13 Hz); PER: 0.01 Hz (−0.16 to 0.18 Hz)]. We further looked at individual cells. In the neocortical sensory areas (S1BF and V2L) and dHC, single-cell firing rate was modulated by theta power for a considerable fraction of phase-entrained cells [percentage of phase-entrained cells modulated by theta power: S1BF: 35% (24–44%); V2L: 15% (9–21%); dHC: 34% (26–40%); mean plus 95% bootstrap confidence intervals]. However, for most phase-entrained cells, the increase of theta power was not paired with an increase or decrease in firing rate. Only very few nonphase-entrained cells in these areas were significantly modulated by theta power [percentage of nonphase-entrained cells: S1BF: 14% (6–23%); V2L: 8% (4–11%); dHC: 6% (0–12%)]. In PER no cells were modulated by theta power. Thus, the effect of firing rate modulation by theta power was strongest in S1BF and dHC, moderate in V2L and absent in PER, and generally more likely to occur in phase-entrained cells than nonphase-entrained cells (see above for nonoverlapping confidence intervals for these respective groups).

In summary, neuronal entrainment by theta oscillations was detected in sensory neocortical areas S1BF and V2L during object discrimination. S1BF and V2L cells appeared to be strongly and selectively phase entrained when objects were being sampled. Similarly, a smaller fraction of cells was phase entrained in PER during object discrimination, whereas a large fraction of hippocampal cells was phase entrained during all behavioral epochs. High theta power was coupled to higher firing rate for about a third of phase-entrained cells in dHC and S1BF (but less or not so in V2L and PER)—an excitatory relationship that was most notable in phase-entrained cells. These findings are in line with the hypothesis that neocortical phase entrainment in the theta band is intensified when there is a high demand for sensory processing and for the comparison between sensory input and object information retrieved from memory, the latter likely requiring interareal communication.

### Phase entrainment in the hippocampus is prolonged and dissociable from firing rate

Our next aim was to explore the finer temporal phase-entrainment dynamics throughout the behavioral trial, elucidating the duration and strength of phase entrainment across the trial and its relationship with the cell's firing rate. We hypothesized that phase entrainment of cells is a neural mechanism different than rate coding (O’Keefe and Burgess, 2005), which we tested by comparing the temporal patterns of phase entrainment and firing rate.

We first characterized the phase distribution of dHC spikes relative to same-area theta oscillations over time (Fig. 7). To do so, we quantified the strength of phase entrainment over time using the PPC and a sliding window (width, 200 ms). We then detected epochs of significant phase entrainment for a cell using random permutation testing (500 shuffles, *p* < 0.01; cells were excluded from this analysis in case of low firing rates or an absence of significant phase entrainment; see Materials and Methods). This method allowed us to measure the onset and offset of phase entrainment in single cells.

The phase entrainment of single dHC cells (*n* = 80 cells) on same-area theta oscillations varied between individual cells, showing a variety of firing behaviors, including cells locking during locomotion (object approach) epochs (Fig. 7*A1*), cells locking before and during sensory sampling (Fig. 7*A2*), and cells locking throughout the full behavioral trial (Fig. 7*A3*). Characteristic for phase-locked dHC cells was their long locking duration (990 ± 389 ms, mean ± SD; Fig. 7*D*; see the next section for significant differences to V2L and S1BF) and the tessellation of all behavioral epochs by the whole population. All task segments were covered by different hippocampal cells (Fig. 7*B*). Phase entrainment was significantly increased compared with baseline during the object approach (*T* = 26; *p* < 0.01; Wilcoxon signed-rank test), sensory discrimination (*T* = 151; *p* < 0.01), reward site approach (*T* = 183; *p* < 0.01), and outcome (*T* = 671; *p* < 0.01) epochs (Fig. 7*E*). Across consecutive behavioral epochs, phase entrainment decreased, that is, phase entrainment was stronger in the object approach versus discrimination epoch, and the discrimination and reward site approach epochs contained stronger phase entrainment than the outcome epoch (*p* < 0.01 for all comparisons; Wilcoxon signed-rank test).

Phase-entrainment dynamics could depend on modulation of a cell's firing rate. For instance, if cells are inactive outside the sensory discrimination epoch, the selectivity of phase entrainment for sensory discrimination would be a result of the absence of firing activity outside the corresponding epoch. To examine whether phase entrainment of dHC cells can be dissociated from fluctuations in firing rate, we next asked to what extent the firing rate was positively correlated with the phase entrainment of cells, as would be expected if PE strength is strongly driven by an increase in firing rate. For a considerable fraction of the dHC cell population (30% of cells), the firing rate was not significantly correlated with phase entrainment (Fig. 7*C*; Pearson’s correlation; *p* < 0.01) versus 31% cells positively and 42% cells negatively correlated (Pearson’s correlation; *p* < 0.01). These data reveal a variety of phase-entrainment dynamics in the dHC cell population: cells are phase entrained for prolonged periods of time, and their firing rate is not a predictor for phase entrainment per se, supporting that individual cells may contribute to changes in synchrony dissociated from firing rate (O’Keefe and Burgess, 2005).

### Phase entrainment in the somatosensory, visual cortex, and perirhinal cortex is transient and selective for sensory processing

Considering the increase in phase synchronization in S1BF, V2L, and PER during sensory discrimination (Fig. 5), we next asked how phase entrainment in these areas dynamically evolves over trial time. We repeated the previous analysis for S1BF and V2L populations (Fig. 8). For this analysis, we used theta oscillations recorded from the neocortical areas (same-area LFP), as we found cells to be more strongly entrained to cortically recorded theta oscillations than dHC recorded oscillations (Fig. 5). We repeated the analysis for PER cells as well, however, due to the low firing rates of PER too few cells reached significance to allow reliable interpretations.

The overall strength of phase entrainment of S1BF and V2L cells on same-area theta oscillations was weaker than that of dHC cells (S1BF: PPC = 9.47 × 10^−3^ ± 2.33 × 10^−3^, mean ± SEM; V2L: PPC = 1.06 × 10^−2^ ± 2.44 × 10^−3^; dHC: PPC = 1.18 × 10^−1^ ± 2.15 × 10^−2^; *p* < 0.01; Mann–Whitney one-sided test). In contrast to dHC, where cells were phase entrained throughout behavioral epochs (including the baseline epoch), neurons in S1BF and V2L were generally entrained in a short window following stimulus onset (Figs. 7*A5*,*B5*, 8*A3*,*B3*; locking durations: S1BF: 187 ± 209 ms; V2L: 180 ± 166 ms, mean ± SEM), significantly shorter than dHC locking durations (*p* < 0.01; Kruskal–Wallis, post hoc Mann–Whitney test).

We further looked into the effects of firing rate modulation on phase entrainment and thus quantified the Pearson’s correlation between a cell's firing rate and phase entrainment. For about half of the cells in both S1BF and V2L, the phase entrainment was not correlated to their firing rate (Fig. 8*A6–B6*; S1BF: 24% positively correlated, 20% negatively correlated, 56% not correlated; V2L: 44% positively correlated, 6% negatively correlated, 50% not correlated; *p* < 0.01). Therefore, the selectivity of neocortical phase entrainment for the sensory discrimination epoch cannot be attributed to modulations in firing rate alone.

Overall, these results show a marked, transient phase entrainment of sensory neocortical cells selectively during object sampling. Interestingly, for large fractions of cortical cells, the phase modulation of spike timing cannot be explained by changes in firing rate, as this parameter was uncorrelated with phase locking in these cells. Thus, phase entrainment in the two sensory cortices is selectively enhanced when the animal is engaged in object sampling, even though cells remain active outside the epochs of sensory discrimination.

### Phase entrainment in the sensory cortices, but not the hippocampus, is selective for stimulus modality

If phase entrainment is a mechanism supporting sensory and mnemonic processing, we expect it to occur preferably during stimulus presentation in a sensory area's conventionally associated modality (i.e., the preferred modality for S1BF being tactile and for V2L visual). For each cell that was significantly phase entrained during the discrimination epoch (Fig. 5; S1BF: *n* = 54 cells, V2L = 85 cells, PER = 35 cells, dHC = 122 cells), we computed the phase-locking strength (PPC) during this epoch (0–500 ms following sampling onset) with the trials split per sensory modality. We asked first whether cells were significantly phase entrained (*p* < 0.01, permutation test) only during tactile trials (tactile-only, not phase entrained during *V* trials), during visual trials (visual-only, not phase entrained during *T* trials), during stimulus presentation in either modality (“both,” phase entrained during both *T* and *V* stimulus conditions), or during multisensory trials only (multimodal-only, no phase entrainment during *T* and *V* trials alone). Using this classification, we excluded cells that were not significantly phase entrained during any of the modality conditions (some cells marked as phase entrained throughout the session were not during the modality conditions due to the lower trial count). We found the largest fraction of cells in sensory areas S1BF and V2L to be phase entrained only during stimuli of the preferred modality of both areas (Fig. 9*A1–B1*; the percentages in this and the following paragraph are estimates of the mean percentage and 99% bootstrapped confidence intervals of modality phase-entrained cells), S1BF: 48% (31–65%) of cells tactile-only, and V2L: 48% (32–61%) of cells visual-only. However, in both areas a smaller but significant fraction of cells was phase entrained selectively for the nonpreferred modality [S1BF: 17% (6–30%) visual-only; V2L: 17% (8–31%) tactile-only].

In PER a significant fraction of cells was selectively phase entrained during tactile trials, but not during visual trials, and vice versa [tactile-only: 50% (21–73%) of cells; visual-only: 25% (4–46%) of cells; both *T* and V: 21% but n.s. relative to 0%]. Such separation of phase-entrainment preference for either modality could be due to separation of modality processing along the rostral-caudal axis of PER (Burwell and Amaral, 1998). In dHC, fewer cells were selective for a single modality [dHC: 13% (6–23%) of cells tactile-only; 15% (7–24%) of cells visual-only] but predominantly during both types of stimulus presentation [dHC: 65% (53–75%) of cells during both stimulus conditions].

We thus find that during visual stimulus presentation, a subset of S1BF and V2L cells is firing in sync with the theta rhythm, while the same set of cells is not firing rhythmically during tactile-only input. Furthermore, another subset of cells is phase entrained only during the presentation of both tactile and visual stimuli. The increase in synchrony of many sensory cortical cells is likely correlated to the engagement of these areas in unimodal processing, although a minority of cells is entrained in the nonpreferred modality.

We further asked whether the selectivity for modality in phase-entrained cells was represented by the locking strength and if the selectivity for modality in the sensory areas could be explained by modulations in firing rate or theta power. Phase-entrained cells in the sensory cortices S1BF and V2L were more strongly phase entrained, as assessed with the PPC measure, during trials of the modality associated with the area under scrutiny, compared with the nonassociated modality (Fig. 9*A2–B2*; area S1BF: *H* = 62, *p* < 0.01, Kruskal–Wallis; post hoc one-sided Wilcoxon's signed-rank test: *T* vs *V*: *p* < 0.01, *T* vs *M*: *p* = 0.02, *M* vs *V*: *p* = 0.02; area V2L: *H* = 10, *p* < 0.01, Kruskal–Wallis; post hoc one-sided Wilcoxon's signed-rank test: *V* vs *T*: *p* < 0.01, *V* vs *M*: *p* = 0.01, *M* vs *T*: *p* = 0.01). Possibly, also the firing rate of these S1BF and V2L cells could be modulated by stimulus modality, which could be a confound in interpreting these findings. To investigate this, we computed the average firing rate of each cell in both tactile-only and visual-only conditions (Fig. 9*A3–B3*). No significant differences in firing rate were detected between modalities for the phase-entrained cells (S1BF: *H* = 0.77, *p* = 0.68, Kruskal–Wallis; V2L: *H* = 2.68, *p* = 0.26, Kruskal–Wallis). As an additional control for the potential contribution of changes in firing rate to the modality dependency of phase entrainment, we recomputed the phase entrainment of S1BF and V2L cells while sampling the same number of spikes between modalities (i.e., the modal condition marked by the higher firing rate was downsampled). Even when correcting for modulations in firing rate in this manner, S1BF and V2L showed stronger phase entrainment in their associated modalities (data not shown; S1BF: *H* = 15, *p* < 0.01; V2L: *H* = 27, *p* < 0.01, Kruskal–Wallis).

A second potential confound being at stake is whether LFP theta power had higher amplitudes in either modality. The modality selectivity of phase entrainment could be due to the absence and presence of theta oscillations during the various stimulus presentations (i.e., phase entrainment increases when theta amplitude increases). To investigate this, we extracted theta oscillation amplitudes at the moment of cell spiking and compared the average spike-triggered theta LFP amplitude between the tactile-only and visual-only conditions for each cell (Fig. 9*A4–B4*). In neither area was the theta power significantly higher during any of the stimulus modalities (S1BF: *H* = 0.42, *p* = 0.81, Kruskal–Wallis; V2L: *H* = 0.81, *p* = 0.67, Kruskal–Wallis). When correlating the spike-triggered theta amplitude and the PPC, a significant correlation was found for V2L cells during the visual-only condition (Pearson’s correlation; *r* = 0.64; *p* < 0.01). No correlation between theta amplitude and PPC was found in the other conditions or in the other areas (Pearson’s correlation; *p* > 0.01). Taken together, these results show that modulations in theta amplitude do not explain transient phase-entrainment sensory cortical cells.

These results highlight that theta phase entrainment in S1BF and V2L largely conforms to unisensory processing, while some S1BF and V2L cells are phase entrained selectively during presentation of non-preferred modalities (S1BF: visual-only, V2: tactile-only) and some V2L cells are phase entrained only during multimodal conditions. PER cells are predominantly selective for a single modality, be it tactile or visual. Locking of dHC was mostly found to be nonselective for these modality conditions.

## Discussion

In our object discrimination paradigm, animals rely on the processing of sensory information, memory recall during object detection, and decision mechanisms to choose a reward site. We recorded neural activity from areas S1BF, V2L, PER, and dHC, detected theta rhythm in all four recorded areas, and found that its power depended on the type of behavioral epoch, increasing during object approach, sampling, and reward site approach epochs across all areas (Fig. 3). Furthermore, we found a strong coherence between the theta-band LFPs recorded from sensory neocortical areas and dorsal hippocampus.

The key results of our study lie in the characterization of the temporal dynamics of phase entrainment of sensory neocortical cells by theta oscillations and its dependency on processing task-relevant features. Phase entrainment of S1BF, V2L, and PER cells was prominent during the behavioral epoch of sensory discrimination (Fig. 9). At the same time, it was not significant during the epochs of object approach, reward site approach, outcome, or ITI and reward delivery, which contrasts with previous findings on theta-band phase locking in rat orbitofrontal cortex (van Wingerden et al., 2010).

The role of theta synchronization in sensory processing is further underlined by the reported modality dependence of cell entrainment: cells were more strongly phase entrained during stimulus presentation in their preferred modality as compared with the nonassociated modality (Fig. 9). The selectivity of phase entrainment of neocortical cells for object sampling contrasts with the phase entrainment of dHC cells, which tessellated the full behavioral task (Fig. 7) and were nonselective for stimulus modality (Fig. 9).

The latter finding aligns with the idea of hippocampal theta as a general reference signal for episodic memory encoding during various behaviors such as locomotion, sensory discrimination, spatial choice, and reward. In other words, the hippocampus may be “open” for extended periods to process theta-phased inputs throughout behavioral epochs, in agreement with its general role in recording lifetime events in episodic memory (Eichenbaum, 2000; Pennartz et al., 2002; Robbe and Buzsáki, 2009; Battaglia et al., 2011). In contrast, S1BF and V2L cells were phase entrained during object sampling for epochs of only a few hundred milliseconds (Fig. 8) and predominantly for their preferred modality.

### Neocortical theta oscillations cohere with hippocampal theta rhythm

The exact origin of theta oscillations in areas S1BF, V2L, and PER in our data remains unclear (cf. Schneider et al., 2021). Neocortical theta oscillations probably result from multiple mechanisms interacting, such as volume conduction from the hippocampus (Sirota et al., 2008; Vinck et al., 2016), polysynaptic projections from HPC cells (operating in association with the medial septum and entorhinal cortex; Kleinfeld et al., 2016) to these neocortical areas, or theta oscillations generated by these cortical areas themselves (Ma and Leung, 2018). Therefore the question of which region is the entraining oscillator for neocortical activity cannot be answered simply.

In the case of perfect volume conduction, the phase entrainment of neocortical cells should be equally strong when referring to either same-area or dHC LFP. We found this was not the case, as fewer cells were phase entrained on dHC LFP than those on same-area LFP (Fig. 5*A2–C2*). Furthermore, we found that most cells were selective for either same-area or dHC LFP, but rarely for both (Fig. 5*A3–C3*). These findings contrast with the hypothesis that theta recorded in S1BF and V2L would be purely volume conducted. The phase-locking preference of neocortical cells for same-area theta oscillations, over dHC-recorded LFP, may arise from the distance between our dHC and neocortical recording sites, in combination with phase differences of theta oscillations across the septotemporal hippocampal axis (Lubenov and Siapas, 2009; López-Madrona et al., 2020) or from hippocampus-independent theta generators in the neocortex and possibly thalamus (Ma and Leung, 2018). Finally, we found significant theta activity in neocortical areas during the object approach epoch, while no theta phase entrainment of spiking activity was found during this epoch. To better understand the origin of the oscillations reported in this work, future experiments can use high-density silicon probes to determine current source density profiles and thus obtain more insight into the laminar origin of the oscillations. Moreover, new inactivation experiments targeting the hippocampus and sensory neocortex are expected to reveal their causal roles in the genesis of neocortical theta rhythms. Thus, even though it is unknown which inputs exactly govern spiking behavior, theta oscillations are likely not generated purely locally during this behavioral epoch; more research is required to examine which factors govern firing behavior in these areas, besides local synaptic input in the theta band.

Elucidation of the origin of sensory neocortical theta activity must await further hippocampal inactivation experiments. Regardless of this origin, neocortical theta activity showed considerable coherence with hippocampal theta (Fig. 3*A3–C3*), and consequently it is warranted to discuss the observed phase locking of neocortical cells in relation to functions of hippocampal theta rhythm.

### Phase entrainment in sensory neocortices is driven by modality-specific sensory input

Previously Grion et al. (2016) described theta phase entrainment in S1BF during a tactile discrimination task. While not recording PER or HC spikes, they found a transient increase of hippocampal theta power during whisking paired with increased phase entrainment of S1BF cells by hippocampal theta and increased synchrony between whisking oscillations and hippocampal theta. On a related note, a mechanism for sensorimotor integration, including bidirectional coupling between the hippocampus and S1BF, was proposed (Kleinfeld et al., 2016; cf. Dorman et al., 2023). In short, before engagement in tactile discrimination, hippocampal theta oscillations may pull whisking-induced oscillations into their theta frequency range. Tactile sensory information may thus be sent through the ascending cortical pathway to the hippocampus.

Our results build on the findings of Grion et al. and add several pieces to the puzzle of corticohippocampal network functioning. First, we assessed phase entrainment of S1BF cells in the absence of a tactile stimulus (i.e., during visual-only trials), while the animal was nonetheless engaged in whisking. In the presence of whisking but in the absence of a tactile stimulus, most S1BF cells were not phase entrained, even if they were significantly phase entrained in other conditions (Fig. 9). Thus, phase entrainment in S1BF (and also V2L) is at least partially driven by sensory input. In addition, a small pool of S1BF cells was phase entrained selectively during visual-only stimuli, demonstrating cross-modal interactions in the theta band (cf. Bieler et al., 2017). Second, in contrast to Grion et al., our task incorporated a significant spatial component, allowing the animal to move around the T-shaped track freely, which likely drove the increased theta power during the approach epoch. While theta power was high during object approach in all recorded areas, phase entrainment in S1BF and V2L remained largely absent (Fig. 8), indicating that an increase in theta power alone is not enough to enhance phase entrainment in these areas. Third, we report a dissociation between rate and phase coding in S1BF and V2L, which implies that phase entrainment in S1BF and V2L may serve a different function than rate coding. Fourth, different from Grion et al., we presented objects in two sensory modalities, which allowed us to establish that stimulus-induced phase entrainment in S1BF and V2L was largely modality specific. Our results substantiate how S1BF, V2L and PER population synchrony increases during the processing of sensory information. Somewhat surprisingly, theta phase entrainment of PER cells was relatively weak relative to S1BF and V2L, which is, however, in line with recent findings on single-neuron to population coupling in the same network during a visual discrimination task set on a figure-8 maze (Dorman et al., 2023).

The behavioral relevance of our findings warrants further research. While Grion et al. did report a positive correlation between behavioral performance and increased whisking theta oscillation synchrony, we were not able to find a behavioral correlate such as faster reaction times with increased phase entrainment (data not shown). This might be due to the relative long duration of our behavioral trials and the self-paced nature of our behavioral task.

### Mechanisms of phase entrainment in sensory cortices

The synchronization of two connected systems by phase entrainment may enable and modulate communication, for instance, during memory read-in and read-out depending on sensory stimuli (for review, see Colgin, 2016; Lakatos et al., 2019). The phase entrainment we describe in sensory cortices thus might act as a neural mechanism which aligns incoming sensory input and cortical population activity, both locally and with respect to remote dynamics in the hippocampus.

**Figure 10.**
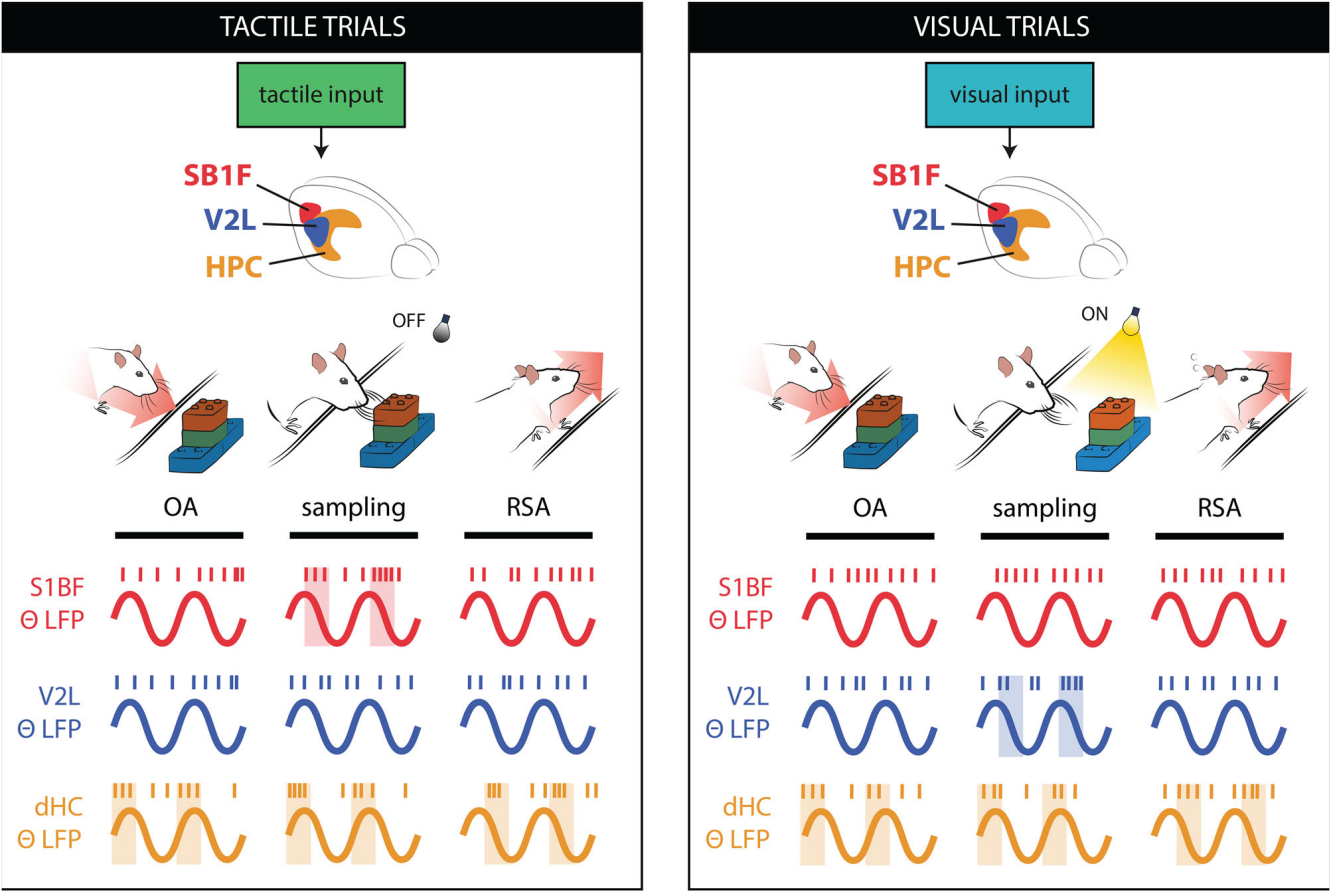
Conceptual summary. Modality-selective theta phase entrainment of cell activity in somatosensory and visual cortices is coupled to a phase advance in hippocampal firing. During the object approach (OA) to the sampling area, S1BF and V2L are minimally engaged in sensory processing, and cell activity in these areas is largely characterized by asynchronous activity. PER is now shown here but displayed similar patterns as S1BF and V2L (albeit with tactile or visual preferences in phase entrainment). In HPC, cells are phase entrained throughout different behavioral epochs. When the rat engages in sensory discrimination, and S1BF and V2L are recruited for sensory processing, cell activity is phase entrained by local theta rhythms, largely selective for the modality preferred by each respective area. This temporal structure may support a role for theta-band activity in neocortical-hippocampal communication, potentially subserving read-in and recall of sensory-mnemonic information.

Phase entrainment of sensory and perirhinal cortex activity occurs primarily during sensory acquisition, while the hippocampal theta rhythm is proposed to serve as a necessary background state for encoding episodic memories during various behaviors (Fig. 10). When the neocortex is not engaged in processing information, its population firing patterns are characterized by asynchronous firing, that is, without consistent phase relationship with theta oscillations. Following stimulus onset, population synchrony increases (as reported by the increase in phase entrainment), due to the rhythmicity of the sensory input, the network's intrinsic oscillatory mechanisms and/or top-down input, for instance, from the medial temporal lobe.

Interestingly, the presence of theta power was not a predictor for phase entrainment per se (Figs. 6, 9). This could, first, be explained by the fact that the recorded oscillations are partly generated outside the area of recording or second, by the finding that theta oscillations were mostly generated by subthreshold membrane potential activity (Noguchi et al., 2023), as theta oscillations could be present without generating spikes. Third, somatic inhibition can veto spiking yet contribute to extracellular current flow and LFP magnitude (Buzsáki et al., 2012). Similarly a single cell's firing rate (Figs. 7, 8) was not predictive of phase entrainment (O’Keefe and Burgess, 2005). These possibilities call for more research on the factors determining whether a cell is phase entrained or not.

The increased synchrony of neocortical cell firing may facilitate and drive information processing in higher sensory and mnemonic areas, possibly through synchronizing interconnected areas by aligning cycles of excitation and inhibition (Colgin, 2016). The phase entrainment in sensory cortices was characterized by its brief duration of only a few theta cycles. Such short-lived phase entrainment nearly contradicts (Lakatos et al., 2019), who define entrainment as requiring rhythmicity. However, evidence has been raised for phase coding based on nonrhythmic, irregular, oscillations (Eliav et al., 2018), and the phase entrainment reported here could thus be explained as spike synchrony relative to the synaptic population activity, rather than depending on sustained oscillations. Following the offset of sensory input, the sensory regions return to an asynchronous state, whereas the hippocampus continues to linger in a theta phase-entrained state, as other event information may need to be processed at any time point in the task.

## Data Availability

The experimental data used in this study is registered as a dataset in the EBRAINS database. A detailed description of the experiment and the included data are published in the EBRAINS knowledge graph: https://doi.org/10.25493/AM91-2D. The code for analyzing these data and generating the figures is publicly available: https://gitlab.com/Truikes/vita-lfp.

## Synthesis

Reviewing Editor: Christine Portfors, Washington State University Decisions are customarily a result of the Reviewing Editor and the peer reviewers coming together and discussing their recommendations until a consensus is reached. When revisions are invited, a fact-based synthesis statement explaining their decision and outlining what is needed to prepare a revision will be listed below. The following reviewer(s) agreed to reveal their identity: Andrew Maurer. Note: If this manuscript was transferred from JNeurosci and a decision was made to accept the manuscript without peer review, a brief statement to this effect will instead be what is listed below.

The authors agree that the manuscript has been improved in this revision. However, some issues remain that should be addressed to improve clarity and provide a clear interpretation of the results. In particular, the manuscript frequently employs the term "synchrony," which traditionally implies zero-phase lag. However, the work of Mizuseki and Buzsaki, among others, suggests a progressive wave of activity across hippocampal regions rather than synchronous firing. This raises questions about the synchronization across various brain regions, considering factors like axonal conduction delay. The reviewers recommend a clearer definition of synchrony as used in this study, particularly in the introductory sections.

Additional comments:

Regarding the theta phase lag analysis, it would be helpful if the authors to provide detailed statistics including average theta phase lags and their deviations. This should be done for different task epochs and in relation to theta power. These analyses are distinct and should be treated as such. A phase lag of 0 degrees with minimal deviation implies synchrony, but the EEG data presented seems to suggest otherwise. Are these phase differences statistically significant compared to a null model, like a shuffle test?

Concerning the WPLI, clarity on what the authors deem as a meaningful level would be beneficial. For example, while 0 indicates complete inconsistency and 1 perfect alignment, how do the authors interpret intermediate values like 0.5? Similar questions apply to the PPC values reported. The low values for S1BF and V2L might be statistically distinct from 0, but their biological relevance remains unclear. Could such values arise from random processes? Further elaboration on the interpretation of these values would be enlightening.

It would also be helpful if the authors delineate the chosen parameters for the wavelet transform, such as the number of cycles. This choice impacts the balance between time and frequency resolution and hence the study's conclusions. What were the considerations behind these methodological decisions?

Regarding the influence of power on coherence, an analysis sorting 1-sec LFP segments by theta power and calculating coherence across deciles would be insightful. This could ascertain if power is the primary factor influencing coherence.

The manuscript discusses how phase-entrainment selectivity might not be solely dependent on the presence of theta oscillations. However, this seems at odds with the understanding that LFP is largely influenced by synaptic transmembrane currents. Could the authors expound on how increased theta power doesn't necessarily lead to greater entrainment?

In this context, it would be valuable to see depth-of-modulation plots comparing neuronal activity across different levels of theta power. This would provide a more nuanced understanding of how neurons respond to varying theta rhythms.

Finally, the absence of a limitations section in the discussion is notable. Addressing potential limitations and alternative interpretations enhances the manuscript's credibility. I recommend the authors include such a section, discussing not only the relationship between power and phase entrainment but also other relevant aspects like the generalizability of findings and methodological constraints.

## Author Response

Reviewer 1 Some suggestions and questions with the current analysis include:

1.1 During the sensory discrimination (object sampling) period, the rats reached their head into the sampling area. Therefore, they are likely to be in a state with reduced motion, compared to approaching and exiting periods. In such quite wakefulness & active sensing period, animals are reported to have low-theta, alpha-like oscillations (M Einstein et al., JNeurosci, 2017). Could the author show some original LFP trace examples during this period, in addition to the power spectrums that were shown? We would like to thank the reviewer for his/her constructive comments.

We initially refrained from adding those plots to avoid redundant data. However, we agree that the original LFP traces are insightful as well and, following this suggestion, we have added them to Fig. 3 (panels A1-D1 in the resubmission).

With regard to the low-theta, alpha-like oscillations mentioned (n.b.; in rodent literature we typically refer to the frequencies below theta as delta): when aligning the data to the point of immobility (when the animal stops moving forward before moving backwards), we indeed do see a low-frequency fluctuation during the period of immobility. We found the power of this peak to be between 0.5-2 Hz. As of to date, we are uncertain what this fluctuation/oscillation exactly is, but we are working on another analysis which should shed more light on this phenomenon. Because these data raise many more questions (e.g. where does the oscillation arise? why does it have only 1 or 2 cycles?) than we can answer at this time, we feel it is better not discuss it in the current work, to prevent this study to become a mixed bag of results with a less coherent narrative.

1.2 How quickly do rats learn this task? Histogram with task accuracy across days will be very helpful, if such quantification exists. How many trials are used in final accuracy rate quantification? Rats learn this task over the course of several months, and in several phases. The training was deemed complete once the animals achieved 70% correct in all modalities for seven consecutive days.

We added this information to the Methods, line 123: "Behavioral training spanned several months and was deemed complete once stable performance of 70% correct for each modality during seven consecutive days was reached." The behavioral performance was stable across recording days. For the sake of completeness, we added an example histogram in Fig. 1D and a reference in the text at line 330: "Behavioral performance was stable across recording days (example data in Fig. 1D)." We have added the trial count in a table (Table 1) and refer to it in line 325: "... (see table S1 for trial count per animal and modality),...) 1.3 Are heat maps for population pairwise phase-consistency (PPC) in Fig. 5B and Fig. 6A3, B3 cross-validated? Are these examples of a single trial corresponding to the same recording location, or they were compiled from different trial and session? Fig. 6B3 looks like V2L neurons have PPC spread throughout the entire period as well.

The heatmaps cannot be cross-validated for this (PPC) analysis because this metric is measured across trials, in contrast to e.g. firing rate. We estimate the significance threshold using permutation tests, and selecting only half of the included trials will increase the variance of the PPC in the permutation testing (Vinck et al., 2021, Bos et al. 2017). The current selection criteria (p < 0.01, for at least 10 consecutive bins) are quite rigorous.

The data in fig. 6B and fig. 7 (previously fig. 5B and 6) were computed over a full session for each neuron, and neurons were sampled from all recording sessions. We extended the caption of Fig. 6, line 910, with: "Only significantly phase-entrained cells, as per this analysis, were included (n=80, sampled across 17 sessions)." The caption of Fig. 7, line 925, now reads: (n=17, recorded in 12 sessions)." And line 934: "V2L (n=17 cells, recorded in 12 sessions)." As regards the apparent PPC spread of some V2L neurons: some V2L cells are indeed not selectively entrained during the sampling epoch. In this analysis we do not pre-select cells based on their phase-entrainment selectivity for a particular (sampling) behavioural epoch. Nonetheless, there is a concentration of V2L cells with their PPC peak in the epoch of sensory sampling.

1.4 Why not include T&V quantification in 2-4 columns like column 1? Otherwise, needs to point out specific reason why not. Not so clear in text from my reading.

The difference between column 1 and columns 2-4 of figure 8, is that column 1 summarizes single-cell phase-entrainment preferences. Thus a cell can be phase-entrained during both the T and V condition. For these plots, we thus label each cell and then summarize the relative counts. However, the remaining columns (columns 2-4) are population aggregates; for each phase-entrained cell we compute the statistic (PPC/FR/theta amp) per modality and then compare between groups. If we were to implement that T&V label in these columns, then, for e.g. panel 8C4, we would first compute theta amplitude during the T condition, then during V condition, and then also a 3rd time while sampling data from all T and V trials. However the latter, viz. the amplitude sampling from all T and V trials together, would contain the same information as the sampling from the individual (T, V) measurements. In summary, the label 'T & V' in in the first column of fig.8 means that the cells are phase-entrained during both the T and V condition in C4, while the other columns describe the population averages per condition (T/V/M).

To reduce confusion, we removed the labels in the caption of Fig. 8 and updated the caption of panel A1 (line 936) with 'Cell phase-entrainment selectivity for modality'.

- 1.5 Figure 7 column 4 shows no significant difference for theta amplitudes across different conditions. Are these compared within the same session? Or compiled across different animals across sessions? My naïve hypothesis for the modality-dependent phase entrainment differences for SB1F and V2L would be that theta LFP amplitudes in SB1F are high in tactile sampling trials and low in visual sampling trials, within the same session of recording. This will result in phase entrainment differences. Can the authors show quantifications to support or oppose such hypothesis? Also it will be great to bring all scatter dots to the front of the plot, as many are currently missing.

The theta amplitudes are compared within the same session. In this analysis, we select all cells phase-entrained for the area's preferred modality. Then we find for each cell the theta amplitude per spike and average the theta amplitude per modality. In the initial submission, we first compared the groups (group depending on panel, for column 2 that is log(PPC) for the T, V and M modality) using a Kruskal-Wallis test and concluded that groups were not significantly different if p>0.01. However, following this revision, we think that the Kruskal Wallis is not allowed, since it assumes independence between groups. The multimodal and unimodal conditions are not independent (the multimodal condition has a tactile and visual component in it). Thus we removed the Kruskal Wallis test and are now using a paired Wilcoxon's test (also within session comparisons) to determine significance.

Based on our data, we do not find a difference in theta amplitude between modalities in areas S1BF, V2L, PER (Fig 8, A4, B4, C4), but do find a difference in dHC between the T and V condition (Fig 8, D4). For the tactile modality we do not expect this per se, as in all modality conditions the animal is whisking, and theta oscillations are likely present in relation to this rhythmic movement. For the theta oscillations in V2L we do not have a prior on whether these oscillations should be reduced during T trials.

To further test the reviewer's hypothesis, we computed the correlation between the PPC and theta amplitude per cell per modality. Here, we do find a significant correlation in area V2L between the PPC and theta amplitude (r=0.65, p<0.01), but not for the other areas or other conditions. We added this information to lines 551-556: "when correlating the spike-triggered theta amplitude and the PPC, a significant correlation was found for V2L cells during the Visual-only condition (Pearson correlation, r = 0.64, p < 0.01). No correlation between theta amplitude and PPC was found in the other conditions or in the other areas (Pearson correlation, p > 0.01). Taken together, these results show that modulations in theta amplitude do not explain transient phase-entrainment of sensory cortical cells." And we added the suggested hypothesis to the main text, line: 546-548: "The modality selectivity of phase-entrainment could be due to the absence and presence of theta oscillations during the different stimulus presentations (i.e. phase-entrainment increases when theta-amplitude increases).

The scatter dots in Figure 8 are outlier datapoints, while the rest of the distribution is conventionally summarized in the box plot.

- Reviewer 2 In this article, Ruikes et al. report theta phase entrainment of S1BF and visual neurons in a sensory input modality specific manner. The authors propose that such phase entrainment coupled with change in hippocampal phase locking facilitates communication between the sensory cortices and the hippocampus.

The behavioral experiment is well designed to probe neural responses to tactile, visual or tactile and visual stimuli. Unfortunately, inadequate neural data and methodological issues leave a substantial gap between the actual results and the conclusions drawn from them.

We thank the reviewer for his/her critical review of our work. We would like to clarify the following before going into the reviewer's remarks in detail. The main aim of the paper is to describe theta-band activity in sensory cortices, and its dependency on stimulus modality (as in the title). We conclude that, predominantly when cells are processing sensory information, cells in sensory cortical areas are phase entrained. To provide context for the neocortical phase-entrainment, we report the coherency between HPC and neocortical LFPs and the phase-entrainment in HPC.

We hypothesize, and that's how we wrote it down, that such theta-band activity may facilitate communication between populations of neurons. This hypothesis is mainly founded on previous work, but we do and did not claim to have shown conclusive evidence here for inter-areal communication. In the revision, the emphasis has shifted away from inter-areal communication, except where this concept is needed to outline the theoretical framework.

- Major concerns 2.1 -L166-85 "In PER we found a near-zero phase-lag compared to dHC, as expected for an area directly connected to dHC (Fiorilli et al., 2021)." Fiorilli et. al. 2021 cite evidence for weak direct connectivity between perirhinal cortex and dHC. The indirect connection via LEC is stronger, but LEC does not show strong theta oscillations during movement, unlike HC and MEC (Deshmukh et al., 2010). Are the authors suggesting that the weak direct connection is sufficient for 0 phase lag between PER and dHC two regions? Even with strong connection between MEC and HC, there is a phase lag in theta between the two.

Fiorilli et al talk about PRC spikes phase locking to HC theta: "The interactions between gating and execution of unitizing operations in PER remain to be investigated, as well as how PER gating and firing may depend on hippocampal feedback, which in behaving animals may be expressed in phase locking of PER neurons to the hippocampal theta rhythm (Ahn et al., 2019; Bos et al., 2017)." This phase locking would suggest coherence of theta and hence a fixed phase relationship between perirhinal cortex and dHC, but it doesn't necessarily mean a near 0 phase lag, as claimed by the authors here.

Based on this, and the next point of discussion, we decided to remove the phase-lag data. See further discussion under 2.2.

2.2 -L186-9 "This difference in phase lags between animals may be explained by specific tetrode placements in dHC, as recordings from dHC in Rat-1 were made from subfield dorsal CA1 and recordings in Rat-2 were from subfield dorsal CA3, and the theta phase of LFPs can shift between subregions of the hippocampus (Petersen & Buzsáki, 2020).". The exact placement of electrodes in each of the regions is critical to understand what is happening here. For example, theta phase reverses around CA1 pyramidal cell layer and MEC layer I/II.

We have added our histological data (Fig. 2), to clarify the recording locations. Indeed, because the theta phase is reversed around the CA1 pyramidal cell layer, we have reversed the CA3 LFP from rat-2, described in the caption), and now also in the Methods (line 209): "LFP data recorded from CA3 in rat-2 were reversed in polarity." Furthermore, this differential placement of dHC electrodes and consequent phase difference in dHC LFPs in the two rats should lead to a similar difference in phase lag between perirhinal and dHC. The data in Fig 2A/B/C3 does not show changes in phase lags between the PER-dHC LFPs in the two rats matching the changes seen in S1BF-dHC and V2L-dHC.

Surely, perirhinal cortex can't simultaneously have near 0 phase lag with respect to two different hippocampal regions with ~ 90 degrees phase lag with respect to one another.

Unfortunately, the manuscript only has data from 2 animals showing different results, leaving open the question of whether the PER-dHC phase lag reported here is reliable (and if so, explain the difference in sensory cortex-dHC phase lag in two rats). As such, the claims made in this section are not substantiated by the data presented.

In fact, the substantially smaller proportion of theta phase entrained neurons in PER compared to other cortical regions as well as the hippocampus belies the claim (L166-85) that PER-dHC 0 phase lag is due to direct (and hence presumably strong) connection between PER and dHC.

We agree that, on the whole, too few data are presented to make reliable claims regarding the exact phase-shift between dHC and the other recorded areas. The main thrust of the paragraph does however still stand; global coherent theta-band activity between S1BF-dHC, V2L-dHC and PER -dHC. We do not claim here to have reported inter-areal communication. We do claim that our results fit the idea that quantification of theta band synchrony can be used as a method to study interareal communication.

The main concern of these results is the reliability, and interpretation, of the reported phase-lag between these areas. Because of the various uncertainties and the limitations conveyed by having two animals, we decided to leave out the phase-lag LFP data (previously in Fig. 5F) as well as the interpretation and suggestions based on these data.

2.3 -L193-7. "As shown in Fig. 2A4-C4, the strength of phase synchronization (WPLI) differed per behavioral epoch, with the strongest WPLI values being reached during approach, sensory discrimination and choice phases (p < 0.001, one-sided Wilcoxon signed rank test). All three neocortical areas showed similar profiles with their LFP signals referenced to dHC." Perirhinal cortex-dHC WPLI does not significantly differ from baseline in any of the epochs.

This needs to be described here, as well as discussed. It is a curious observation, given that PER, just like all other regions, shows increased theta during multiple epochs compared to baseline. This observation also contradicts the statement, "Taken together, these results show a global presence of coherent theta oscillations throughout the hippocampus and neocortex during task engagement by the animal (Fig. 2)." (L197-8).

WPLI amplitudes in PER-dHC seem smaller in theta range compared to other regions. This observation again contradicts the earlier claim of short phase lag in PER-dHC theta being caused by direct connection between PER and dHC (in contrast to the other two regions and dHC).

It is quite possible that the lower WPLI between dHC and PER is due to the 0 phase-lag. Because the WPLI measure assumes that a 0 phase-lag is volume conducted, it reduces the contribution of 0 phase-lag coherency to zero. Thus, we would indeed expect the WPLI in fig. 3C4 to be lower than between other areas.

As pointed out above in pt. 2.1, we have weakened our suggestion that PER-dHC theta phase relationships would be due to a direct connection, although the demonstrated anatomic presence of this connection is compatible with having such relationships.

The WPLI between neocortical areas (incl. PER) and dHC is significant during all behaviours, we added this to line 365: "We found significant phase synchronization during all behavioral epochs ... of the neocortex (p < 0.05, permutation test) and those ...".

2.4 -L228-9. This observation also goes against the earlier claim of PER-dHC phase delay being short due to direct connection between the two. It also appears that S1BF has more theta modulated cells, and cells phase locked to dHC theta than PER - this needs to be confirmed statistically (compare all regions with each other).

This again shows that PER is less involved in theta dynamics as well as theta mediated synchrony with theta than sensory cortex shown here, contradicting the claims made by the authors of all cortical areas being involved in theta mediated synchrony with HC.

In the sentence hereafter (line 412) we wrote: "Thus, we did find sparse phase-entrainment in PER, which did not preferentially occur with respect to dHC or PER LFP." Besides our work, there are other studies that back up the claim that PER is involved in theta mediated synchrony with HC (Ahn et al, 2019), thus we feel this statement is justified.

We want to refrain from describing all area-area comparisons in the main text, as this would decrease the readability. Still this information might be of interest to the reader, so we present the exact percentages and estimated confidence intervals in Tables 2 and 3. One can consider fractions significant from each other, if the confidence intervals do not overlap. Coming back to a statistical comparison of phase-entrainment of S1BF vs. PER cells based on these data, we do not see a higher fraction of S1BF cells phase-entrained to dHC LFP than in PER (n.b. the fraction of phase-entrained cells in PER increased following our updates following this reviewer's first minor concern; see pt. 2.22 below).

2.5 -L359-60. "The overall strength of phase entrainment of S1BF and V2L cells on same-area theta oscillations was weaker than of dHC cells". While this population level difference in different areas is informative, the authors need to separately analyze the cortical neurons (in S1BF, V2L as well as PER) that are strongly modulated by theta in greater detail.

For example, do these neurons show longer entrainment comparable to dHC neurons? Do these neurons show entrainment in multiple behavioral epochs? Do these neurons show stronger correlations with hippocampal single neurons or multiunit activity compared to the others? Figure 6 and 7 are intended to do exactly this. Instead of segmenting the data into epochs, we compute the phase-entrainment (PE) over time. This analysis gives us insight into the extent to which cells are PE (phase-entrained) during single or multiple epochs. Indeed in HC, cells are PE during multiple behavioral epochs. In S1BF and V2L cells are predominantly PE during sensory discrimination. Further we examine the duration of phase-entrainment in these areas, as written in the text: (line 481-485): "In contrast to dHC, where cells were phase-entrained throughout behavioral epochs (including the baseline epoch), neurons in S1BF and V2L were only entrained in a short window following stimulus onset (Fig. 7A3 and B3; A5 and B5; S1BF: 187 {plus minus} 209 ms, V2L: 180 {plus minus} 166 ms, mean {plus minus} s.e.m.), significantly shorter than dHC locking durations (p < 0.001, Kruskal Wallis, post-hoc Mann Whitney test)." We now emphasize that these data pertain to locking durations by adding "locking durations" to line 483: "neurons in S1BF and V2L were generally entrained only in a short window following stimulus onset (Fig. 7A3 and B3; A5 and B5; locking durations: S1BF: 187 {plus minus} 209 ms, V2L: 180 {plus minus} 166 ms, mean {plus minus} s.e.m.), significantly shorter than dHC locking durations (p < 0.001, Kruskal Wallis, post-hoc Mann Whitney test).

The suggested correlation analysis could be insightful in principle, but we have too few data to do such an analysis (we have too few cells in a single session to compute reliable pairwise cell-cell correlations). For a study of the same brain system with single-cell to population coupling, see Dorman et al. (2023).

The authors have motivated this study using the need to understand task dependent functional communication between different regions of the brain. The authors argue in the discussion that the lack of entrainment to dHC theta in majority of the neocortical neurons could be because they could be entrained to theta in a different part of the hippocampus. It is also possible that neocortical theta is not correlated with theta in any part of the hippocampus strongly enough to facilitate interareal communication. Alternatively, it is possible that only a subset of cortical neurons effectively communicate with the hippocampus - performing the analyses suggested above will enable the authors to go beyond showing entrainment to local theta, which is insufficient to drive inter-area communication.

We are afraid that the reviewer has misunderstood our motivation for our study and apologize if our writing gave rise to this confusion. We motivated the study through the need to understand changes in theta band activity (not functional communication between different regions of the brain) across task phases and in different, connected brain regions. As other work postulates that theta band activity supports interareal communication, we expected theta band activity in the recorded areas during sensory processing.

The aim of our work is stated in the abstract (lines 11-16) and introduction (lines 65-77). In neither of these sections do we write that our aim is to elucidate functional communication between areas.

2.6 -L146-7 "For each session, we selected one lead per recorded area per session (areas S1BF, V2L, PER, dHC, see Methods)". Were the recording layers in each of the cortical regions the same for all rats? dHC LFP recordings are reported from CA1 for one rat and CA3 for the other. Where exactly in CA1 and CA3 were these recordings from? We mainly recorded from deeper layers (5/6) in all of the neocortical areas. Their recording location was tracked using electrophysiological profiling, depth estimation through the recording device (number of screw turns) and post-mortem histology. This does not mean, however, that the layers could be determined exactly during all recordings (which is also a reason why the phase lag data previously in Fig.2A3-C3 (i.e. Fig.2A3-C3 from the previous version) are hard to interpret).

Per the reviewer's comments (also point 2.2) we have added the histology data (Fig. 2), with details on CA1 and CA3 recordings.

2.7 -Fig 2. The authors need to show sample LFP traces (in time domain) for all regions in different epochs. Fig2 A3, B3, and C3 need to be generated after segregating the data into different epochs, to see if the phase relationships are different in different epochs.

See answer at point 1.1; we have added example LFP traces in Fig. 3A1-D1. Panels in Fig.2, A3-C3 (previous version) were removed (see above). We had separated the data per behavioral epoch, but found no differences between behavioral epochs. For readability, we retain the pooled data, as the split per behavioral epoch and rat becomes unreadable and the split itself does not reveal new information.

2.8 -Statistics are often incompletely reported: mean +/- SD, value of the observed statistics, number of samples/degrees of freedom, whether the differences remain significant after correction for multiple comparisons etc. are missing from multiple places.

We added statistics in tables 1, 2 and 3.

In the methods, we included the section 'Multiple comparison correction' (line 204), stating that all results are Bonferroni corrected, except for the permutation testing: "Reported results are corrected for multiple comparisons using Bonferroni correction, except for the surrogate permutation tests (bootstrap estimation and permutation shuffling), in which case an alternative to cluster-based correction was used." 2.9 -Since there are multiple sessions from each rat, there is repeated sampling, which inflates the degrees of freedom and hence statistical significance. Do all the statistical results remain significant after removing repeat sampling? We do indeed record multiple sessions from each rat, but before most of the recording sessions the tetrodes were lowered, and data is gathered from a new population of cells, in all areas. Thereafter we determine significance on a single cell basis, in which case one cannot correct for repeated sampling.

2.10 -Fig 1. Authors need to show actual sections with tetrode tracks with examples of recording locations in different areas targeted. Authors also need to show a summary figure with all the included recording sites from all rats.

See answers to points 2.2 and 2.6, and suppl. Figures 2 and 3.

2.11 -L154-6. There appears to be a mean/peak frequency difference between outcome (and baseline) and other epochs. Please analyze that statistically and discuss the significance of change in theta frequency in different epochs.

Per the reviewer's request we analysed the theta peak frequency shift. We found the shift to be significant increased (during maximum frequency, around 500ms following stimulus onset, vs theta frequency before stimulus onset) in all areas (non-overlapping bootstrapped 99% confidence intervals) We inserted additional information to the text as follows (lines 351-356): " The stimulus onset was paired with a shift in the theta peak frequency in areas S1BF, V2L and dHC (Fig. 3A2-D2 and A3-D3, non-overlapping bootstrapped 99% confidence intervals). The onset of the frequency shift nearly coincided with the onset of stimulus and reached its maximum around 500 ms following stimulus onset. Theta frequency has been shown to depend on acceleration in body motion (Kropff et al, 2021). This likely explains the shift detected here, as the animals slow down their movement when approaching the object, and afterwards accelerate again." 2.12 -L192-3. Authors need to perform pairwise comparisons between the cortical areas during different epochs also.

Following this request, we compared the WPLI measured between cortical areas and dHC against each other. Both the Friedman test and post hoc tests were not significantly different between the pairs V2L vs S1BF, V2L vs PER and S1BF vs PER (p > 0.01, MCC corrected; all with respect to dHC LFP). We added this on line 371: "and no significant difference between areas was found (p > 0.01, Friedman test)." 2.13 -L248-51. These statements equate theta entrainment in dHC with involvement in task performance. Given that hippocampus is known to show strong theta entrainment during movement and attention, the reasoning used here would imply hippocampal involvement in every task performed by the animal while moving or paying attention (e.g., respiration).

We stated (line 414-416): "Given its role in episodic and spatial memory, the hippocampus is likely involved in at least some of the behaviours required to perform the task (e.g., object discrimination and recognition, spatial choice and navigation)." This does not imply that HPC or its phase entrainment is involved in every task performed by the animal. Therefore we see no clear reason to change the text.

2.14 -L293. Why is this number (80dHC cells) lower than the number of phase entrained dHC cells reported earlier? Fig. 6A/B3: Why are only 17 cells each included in 6A3 and in 6B3? About a third of 167 SB1F and a fifth of 371 V2L cells are entrained to local theta during sensory discrimination (L199-227); "For each cell that was significantly phase entrained during the discrimination epoch (Fig. 4; S1BF: n = 54 cells, V2L = 70 cells, dHC = 145 cells)," For this particular analysis, we increased the constraints for inclusion. We computed the PPC per time bin (200 ms X n trials) which yielded lower sample sizes, which affected the significance threshold we computed using permutation tests (with fewer spikes, the variance in random computed PPC increases). To compensate for an increased false positive rate, we also required cells to be significantly phase entrained in multiple consecutive bins, as a single significant bin may be meaningless (window step size=10 ms).

This method is explained in the methods section: Temporal dynamics of phase-entrainment.

2.15 -L324-5. "The preferred theta phase of dHC cells shifts between behavioral epochs". Was there a CA1 vs CA3 difference? What was the theta phase preferred by CA1 and CA3 neuron populations in different epochs? The significance of changes between epochs described later (fig 5F) can be better understood in the context of answers to these questions.

We investigated whether there were differences between the phase shift in CA1 and CA3. Indeed we found different patterns, in CA1 a significant shift was detected between the sampling and approach epoch only, while in CA3 a significant shift was detected between the outcome and choice epoch only. Because we have data from only one animal per subarea in dHC, we cannot draw reliable conclusions from these data, and we removed the phase-shift results from the paper. Specifically, we removed panel fig 5F (i.e. 5F in the previous version), updated Fig 9 (current version) and removed the findings from the main text (Results & Discussion).

2.16 -L341-3. "If synchronization of neural activity by theta oscillations serves interareal communication, we expect the increase of phase-entrainment in the neocortex during sensory discrimination (previous section, Fig. 4A) to be paired with a change in phase dynamics in dHC". But Figure 4 shows that the increase in phase entrainment is specific to local theta, and not hippocampal theta. This leaves the question of how the different areas communicate open.

Indeed we do not (and do not intend) to make any strong claims on interareal communication with these data. While we do suggest a role of theta-band activity for interareal communication (based on existing theoretical frameworks for theta as an interareal means of communication), we do not claim any of our results provide evidence for such a role on their own. Consequently, in the revision we focus more on the dependence of phase entrainment on task epochs, the differences between areas and the role of sensory modalities.

2.17 -L442-3. "We thus find that during visual stimulus presentation, a subset of S1BF cells is firing in sync with the theta rhythm, while the same set of cells is not firing rhythmically during tactile-only input." Are these cells better entrained by V2L or dHC theta during these trials? We investigated this question by measuring the phase-entrainment (PE) for 'crossmodal cells' (cells that are PE on the non-preferred modality for an area; in S1BF those are cells PE during V trials and in V2L those are cells PE during T trials) on dHC and V2L (in case of S1BF cells) and dHC and S1BF (in case of V2L cells) LFPs.

In S1BF we found 8 (13%) crossmodal cells. PE for these cells was stronger when measured on dHC LFP compared to V2L LFP and S1BF LFP. This PE on dHC LFP was not significant for the majority of cells (permutation test, p > 0.05), only for 2 S1BF cells the measured PE was significant (permutation test, p < 0.05). In V2L we found 11 (=17%) crossmodal cells. For 6 cells we did not have S1BF or dHC LFP. For 1 V2L cell the measured PE, measured against V2L LFP, was significant (permutation test, p < 0.05).

Thus we (1) have too few data to draw strong conclusions on whether crossmodal cells are more strongly PE by dHC LFP than the other sensory area LFP; 2) do find for a very small fraction of the cells that they are more strongly phase-entrained by dHC LFP (and not PE by LFP from the other area).

Given the low cell count, and lack of significant results, we decided to omit these results from our report.

2.18 -L443-4. "Furthermore, another subset of cells is phase-entrained only during the presentation of both tactile and visual stimuli." Are these cells better entrained by dHC theta or by theta from the other regions (i.e. V2L for cells from S1HF and vice versa)? If dHC theta preferentially modulates multimodal and crossmodal representations in sensory cortices, it would argue strongly in favor of theta facilitating communication across different regions of the brain.

We repeated the same analysis as in point 2.17; and found no multimodally enhanced cells to be phase entrained by dHC LFP (nor the LFP of the other sensory area). Also in these groups the cells counts are low (9 for S1BF and 9 for V2L), thus we refrain from drawing conclusions from these data.

2.19 -L477-80. "we found a strong coherence between the theta-band LFPs recorded from sensory neocortical areas and dorsal hippocampus. The key results of our study lie in the characterization of the temporal dynamics of phase-entrainment of sensory neocortical cells by theta oscillations and its dependency on processing task-relevant features." The relationship between strong coherence between theta band LFPs in sensory neocortex and dHC and phase entrainment of sensory neocortical cells by theta is not examined at all in this paper.

We did not intend to claim this, and to avoid the impression that we made such a claim, we have now split the paragraph at (line 596) 'the key results...', to separate the two findings in the text.

2.20 -L517-9. "Regardless of this origin, neocortical theta activity showed considerable coherence with hippocampal theta (Fig. 2), and consequently it is warranted to discuss the observed phase-locking of neocortical cells in relation to functions of hippocampal theta rhythm." Not if most of the sensory cortical cells are not modulated by HC theta.

We disagree with the reviewer; if the neocortical LFPs are coherent with the dHC LFP (see WPLI results, Fig. 3), then the conclusion is warranted that theta-band activity (phase-entrainment of cells by theta) in neocortical areas is at least to some extent related to HPC LFP. Moreover, we found modulation by HC theta of sensory cortical cells (lines 394-407).

It is unclear what mechanism drives this coherence (as discussed), but if these LFPs are coherent in theta-band, then we see a good cause to discuss theta-band activity in one area with LFP theta band in the other.

2.21 -L564-8. Authors need to explicitly test this hypothesis by showing the phase relationships between the cortical neurons and the hippocampal neurons in different epochs.

In this final section of the Discussion we provide context and interpretation of our results. We only stated, as a discussion point: "the increased synchrony of neocortical cell firing may facilitate and drive information processing in higher sensory and mnemonic areas [....]". We have not stated at any point in the paper that we demonstrated synchronization of two connected systems. The cited literature consists of extensive reviews to provide a theoretical framework. We feel the discussion point is thus justified here, as it is merely a point of discussion and is phrased carefully ("may facilitate....").

Regarding the suggested analysis, see point 2.5.

- Minor concerns 2.22 -L395-7. "For each cell that was significantly phase entrained during the discrimination epoch (Fig. 4; S1BF: n = 54 cells, V2L = 70 cells, dHC = 145 cells), we computed the phase-locking strength (PPC) during this epoch (0 - 500 ms following sampling onset)" This approach excludes cells that fail to show significant entrainment over all modalities but have significant entrainment in a single modality. The authors need to include all cells that showed significant entrainment to any modality.

We thank the reviewer for this valuable suggestion. We repeated the analysis, but computed phase entrainment for cells during the sampling epoch now per modality. Indeed we now find higher fractions of cells phase-entrained, and figure 4 and 7, and several paragraphs, are updated accordingly. "We added lines 384-388: Phase-entrainment was quantified during each behavioral epoch, and during the sampling epoch separately for each modality condition. In the following sections, we consider a cell phase-entrained if it is phase-entrained during at least one of the three presented stimulus modalities (we further expand on modality selectivity in the final paragraph of the results section)."- We updated the reported percentages in the text accordingly.

Interestingly, using this approach, we now find unimodally selective cells in PER (updated Fig. 8, C-row). Previously, we would only include cells which were PE when measured across all trials in this analysis (and then compute significance per modality). Now we include also cells which reach significance when measuring during a specific modality.

2.23 -L373-5. "the phase modulation of spike timing cannot be explained by changes in firing rate, as this parameter was mostly uncorrelated or anti-correlated with phase-locking." Negative correlation implies that the phase modulation of spike time is explained by firing rate changes even if it is in direction opposite to that expected by the authors. This conflation of negative correlation with lack of correlation is done in multiple places in the manuscript, and needs to be corrected everywhere. There's sufficiently large proportion of truly uncorrelated neurons to support the argument made by the authors, so there's no need to inaccurately pool negatively correlated neurons with uncorrelated neurons.

We updated our report on this topic to (lines 463-466): "For a considerable fraction of the dHC cell population (30% of cells) the firing rate was not significantly correlated with phase-entrainment (Fig. 5C, Pearson correlation; p < 0.01), versus 31% cells positively and 42% cells negatively correlated (Pearson correlation; p < 0.01)...".

And line 493: "...the phase modulation of spike timing cannot be explained by changes in firing rate, as this parameter was uncorrelated with phase-locking for a considerable fraction of cells".

2.24 -L542-4. "While theta power was high during approach in all recorded areas, phase entrainment in S1BF and V2L remained largely absent (Fig. 6), indicating that an increase in theta power alone is not enough to enhance phase-entrainment in these areas." This argues against local generation of theta. If theta is locally generated during approach, local cells need to be entrained to theta.

This makes sense, although we also note that PE in spiking behavior may also be driven by other factors than the predominantly observable rhythm, such as other synaptic inputs than those underlying theta rhythm, and neuromodulators. We have incorporated the argument to our discussion in lines 603-608: "Finally, we found significant theta activity in the LFP of neocortical areas during the approach epoch, while no theta phase-entrainment of spiking activity was found during this epoch. Thus, even though it is unknown which inputs exactly govern spiking behavior, theta oscillations are likely not generated locally during this behavioral epoch.

2.26 -L225-6. Authors should report stats in a consistent manner: here p is reported as <0.01 (with two places after decimal) in one case, and = 0.004 (with three places after decimal) in another.

We opted to report all p values at two places after decimals, and updated the p values in the report accordingly.

2.27 L106-101. Was there any difference between performance in tactile and visual trials? What was the statistical test for significance used to claim that the observed correct responses were significantly higher than the chance level? Please report all the stats for the test.

Performance during probe trials was indistinguishable from chance level, and we compared performance per modality against probe trial performance. We added the statistics for T and V differences as well:

Lines 326-329: "while performance between unisensory modalities did not differ significantly (T vs. V: t(24) = -0.85, p = 0.40, paired t-test). For all modalities, performance was significantly higher than during Probe trials (51% correct response rate during Probe trials; chance level = 50%, T vs. P: t(20) = 3.87, p < 0.01, V vs. P: t(20) = 3.75, p < 0.01, M vs. P: t(20) = 6.81, p < 0.01, paired t-test)." 2.28 -Authors define choice period as being -1 to -0.5 s relative to reward poke. What happens if this definition is changed to 0 to 0.5 s from withdrawal from sampling area? Surely, the choice is better tied to the time the animal makes up its mind and ends sampling, than -1 to -0.5 s relative to reward poke.

Indeed the animals make their decision likely (internally) while they are in the sampling area, as we can predict the response from their head movement while the animal is in the sampling area (data not reported). The aim of this epoch was not to capture decision making, but to contrast phase-entrainment during sampling with the epoch following the exit of the sampling area.

We updated the 'choice' epoch to reward site approach' (RSA), and 'approach' to 'object approach (OA)' in the paper.

2.29 -L102-3 What happened in the trials when the animals withdrew their heads in less than 0.5 s from the sampling area? Were they excluded? We measured the onset of 'retraction', which is when the animal starts moving away from the object. Trials where retraction was < 200 ms were excluded from the analysis. We added this information to lines 321:

Trials wherein the animal started withdrawing from the sampling area before 200 ms were excluded from analysis.

And line 137: (trials wherein the animal started withdrawing their head before 200ms were excluded from analysis).

Trials wherein the retraction was in the interval 200-500 ms were included, as the animal is still a few hundreds of ms in the sampling area following the onset of retraction.

2.30 -Objects need to be described and their images shown in Fig 1.

We described the objects in line 615 of the previous version (line 101 in current version). We have added images to panel Fig. 1C.
